# The utility of nuclear magnetic resonance spectroscopy in assisted reproduction

**DOI:** 10.1098/rsob.200092

**Published:** 2020-11-04

**Authors:** Gitanjali Asampille, Aswathi Cheredath, David Joseph, Satish K. Adiga, Hanudatta S. Atreya

**Affiliations:** 1 Department of Clinical Embryology, Kasturba Medical College, Manipal, Manipal Academy of Higher Education, Manipal 576104, India; 2 NMR Research Centre, Indian Institute of Science, Bangalore 560012, India; 3 Solid State and Structural Chemistry Unit, Indian Institute of Science, Bangalore 560012, India

**Keywords:** assisted reproductive technology, nuclear magnetic resonance spectroscopy, metabolomics, systems biology, *in vitro* fertilization

## Abstract

Infertility affects approximately 15–20% of individuals of reproductive age worldwide. Over the last 40 years, assisted reproductive technology (ART) has helped millions of childless couples. However, ART is limited by a low success rate and risk of multiple gestations. Devising methods for selecting the best gamete or embryo that increases the ART success rate and prevention of multiple gestation has become one of the key goals in ART today. Special emphasis has been placed on the development of non-invasive approaches, which do not require perturbing the embryonic cells, as the current morphology-based embryo selection approach has shortcomings in predicting the implantation potential of embryos. An observed association between embryo metabolism and viability has prompted researchers to develop metabolomics-based biomarkers. Nuclear magnetic resonance (NMR) spectroscopy provides a non-invasive approach for the metabolic profiling of tissues, gametes and embryos, with the key advantage of having a minimal sample preparation procedure. Using NMR spectroscopy, biologically important molecules can be identified and quantified in intact cells, extracts or secretomes. This, in turn, helps to map out the active metabolic pathways in a system. The present review covers the contribution of NMR spectroscopy in assisted reproduction at various stages of the process.

## 1. Introduction

Infertility is a highly prevalent global health condition affecting approximately 15–20% of individuals of reproductive age worldwide. The major causes of infertility in women include anovulation, anatomical problems and endometriosis, whereas sperm disorders are the important causes of male infertility. Several lifestyle factors such as stress, obesity, urbanization-based work pressure and sexually transmitted infections can impair the fertility of the individuals [1,2]. Assisted reproductive technology (ART) has played an important role to partially overcome the low natural reproductive ratio [3]. Over the past 4 decades, ART has revolutionized the treatment of infertility, and more than 8 million live births have taken place since the first successful implementation of *in vitro* fertilization (IVF) in 1978 [4,5]. The demand for ART has increased significantly in recent years; using which a large number of successful pregnancies have been achieved [3,6].

The ART fertility clinic success rate report of the USA showed that a total of 284 385 cycles of ART were performed in 2017 [6]. Age-dependent variation is seen in the type of ART cycles (non-donor or donor oocyte cycle) [3,6,7] and the live-birth rate is inversely related to the patient age [3,7]. As per the 2016 registry of the European Society of Human Reproduction and Embryology (ESHRE), after treatment with IVF and intracytoplasmic sperm injection (ICSI), the clinical pregnancy rates per transfer were 34.8% and 33.2%, respectively [8]. Multiple pregnancies pose a potential health risk for the mother, including pregnancy-induced hypertension and postpartum haemorrhage [9]. Hence, several countries (such as Finland, Sweden, Belgium, Australia and New Zealand) have employed elective single embryo transfer to reduce the possibility of multiple gestation and eventual complications [10,11]. However, many countries are yet to adopt the strict regulations in ART practice, presumably due to the expense involved coupled with the patient's hope to achieve pregnancy in each cycle with better chances. In general, the transfer of multiple embryos is primarily driven by the number of available embryos and overall embryo quality. Thus, the selection of an ideal gamete or embryo that results in a higher success rate together with reduced multiple gestations is the prime focus of ART today, and any improvements in the existing methods for preimplantation embryo assessment would be beneficial for this purpose.

Morphological evaluation is the conventional approach used to select a healthy embryo for transfer as it is considered to be safe, specific and simple [12]. However, this static mode of evaluation has drawbacks, such as subjectivity of the evaluator, frequent assessment during embryo development and culture stability [13]. Evaluation efficacy has been enhanced with the recent development of time-lapse imaging systems allowing embryologists to perform a non-invasive and dynamic evaluation of embryo quality in the steady culture environment [14,15]. In addition, an important observation has been made about the uptake and release of metabolites by sibling human embryos, having identical development and morphology showing significant differences, which could be attributed to genetic and functional heterogeneity in gametes [16,17]. The metabolic activity of the embryo has been found to be an indicator of its viability [18]. Hence, it is considered important to assimilate the metabolic profile of the embryo in the selection process to predict its implantation potential. It provides specific metabolic markers to identify competent embryos. Thus, understanding the metabolism of embryos is central to ART.

In recent years, different analytical techniques have been used in ART for studying embryo metabolism. These include near-infrared spectroscopy [19], Raman spectroscopy [20], mass spectrometry (MS) [21] and nuclear magnetic resonance (NMR) spectroscopy [22,23]. NMR spectroscopy is one of the widely used methods for identification and quantification of molecular species in a sample and is an alternative to MS, which is another popular technique used in metabolomics [24–28]. NMR is quantitative and does not require special sample preparation approaches like separation or derivatization that is needed for MS. However, compared with liquid chromatography-coupled mass spectrometry (LC-MS) or gas chromatography-coupled mass spectrometry (GC-MS), NMR is often 10–100 times less sensitive, and NMR-based metabolomics study usually provides reliable information on metabolites with concentrations greater than 10 µM [25].

The present review covers the contribution of NMR spectroscopy in assisted reproduction at various stages of the process. The general principles of NMR in metabolomics are first introduced, followed by its application to various procedures in ART. Apart from this, NMR plays a significant role in understanding the pathophysiology of polycystic ovarian syndrome (PCOS) and endometriosis, which are known to impair fertility in reproductive-age women [29,30]. NMR studies performed on animal models are also included as they have helped in probing the embryo directly and provided translational knowledge, which is not possible in human embryos due to ethical restrictions [31].

## 2. NMR spectroscopy in metabolomics

NMR has emerged as one of the important analytical techniques used in metabolomics. Despite its lower sensitivity, NMR spectroscopy offers many unparalleled advantages over other analytical techniques for studying the metabolic profile of a system [32–35]: (i) the NMR spectra obtained are highly reproducible [36]; (ii) the non-destructive nature of NMR analysis allows repeating experimentation and the sample can be recovered and stored for a long time and used for other studies [37]; (iii) NMR spectroscopy is inherently quantitative as the signal intensity is directly proportional to the metabolite concentrations and the number of particular nuclei in the molecule [38,39]; (iv) NMR can be used for both targeted and untargeted analyses of metabolic flux both *in vitro* and *in vivo* [40]; (v) its inherently quantitative nature enables precise quantification of precursors and products [41]; and (vi) it is well suited for studying intact tissues, organs, and other solid or semisolid samples through solid-state NMR (ssNMR) and magic-angle spinning (MAS-NMR) [42]. However, NMR also has its disadvantages, with the most significant challenge being its lack of sensitivity. In recent years, the development of ultra-high-field NMR spectrometers of 1 GHz and above [43], and advancements made in NMR probes, have greatly improved the application of NMR in metabolomics. Cryogenically cooled probes can increase signal sensitivity by a factor of 3–4. In addition, microprobes or microcoil probes (1 mm TXI and 1.7 mm TXI probes) not only enhance sensitivity but also reduce the required sample size down to a few microlitres [44–47].

ART in humans is a multistep process involving the superovulation of the ovaries through gonadotropin administration to the female partner followed by the surgical aspiration of oocytes which are suspended in the follicular fluid (FF), within the ovary. Oocytes surrounded by the granulosa and cumulus cells are assessed for their maturity and prepared for fertilization. Parallelly, healthy spermatozoa are separated from the seminal plasma of the ejaculate, and fertilization is achieved. Resulting embryos are developed in the laboratory using appropriate culture media, under a controlled environment for 3–5 days. Morphologically normal embryos that followed a developmental timeline [48] are selected for transfer to the patient's womb.

**Figure 1.**
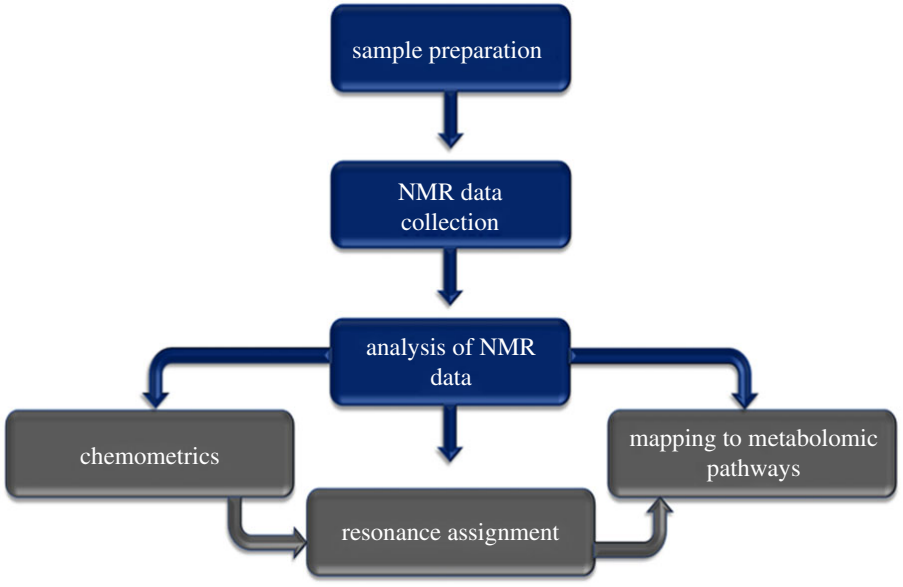
Schematic illustration representing the key steps involved in an NMR-based metabolomics study.

NMR-based metabolomics study generally has three major steps as depicted in figure 1: (i) sample preparation, (ii) recording NMR spectra from the sample, and (iii) analysis of these data to identify metabolites, biomarkers and biological pathways.

The first step in the NMR-based metabolomics involves the collection and preparation of the sample. The volume of the sample needed for NMR data collection is 10–600 µl depending on the probe and the sample tube used. With the advent of low volume and high-sensitivity NMR probes, it is possible to reduce the sample volume even to 5 µl as mentioned above [44–47]. In most of the studies, the sample collected from the source can be directly used for NMR analysis after adding a small amount of deuterated solvent [35]. However, in cases where the sample contains macromolecules such as proteins and lipids, the large difference in the size of the macromolecule and small molecules of interest hamper the analysis due to significantly broadened NMR peaks, resulting in low accuracy of quantification. It is therefore necessary at times to remove high-molecular-weight molecules from the samples. There are two approaches to address this problem. One is to physically separate the macromolecules from the small molecule metabolites using ultra-filtration, solid-phase extraction or protein precipitation using organic solvents such as methanol, acetonitrile, acetone, perchloric acid and trichloroacetic acid [32]. An alternative is to allow the macromolecules to be present in the sample but suppress their signals in the spectrum by special NMR techniques. This is described below while discussing the NMR methods.

The second step is NMR data collection, where the primary goal is to identify and assign the metabolites present in the sample; the metabolites are identified based on their signature pattern in the NMR spectrum. This is usually accomplished using the one-dimensional proton NMR experiment and a set of homonuclear and heteronuclear two-dimensional (2D) NMR experiments. A detailed description of the theory of NMR experiments is beyond the scope of this review. The reader is encouraged to refer to articles on the basics of NMR [49–51]. In certain cases, it is not necessary to remove the protein component from the sample (as alluded to above). The signals from the proteins can be suppressed using specific NMR experiments. Typically, one-dimensional (1D) NMR spectra are recorded using a ‘T2 filter’ procedure, which attenuates the signal from macromolecules such as proteins and lipids [52,53]. One potential drawback of not removing macromolecules from the sample is that it can affect the quantification of small molecules that interact with the macromolecules [54].

**Figure 2.**
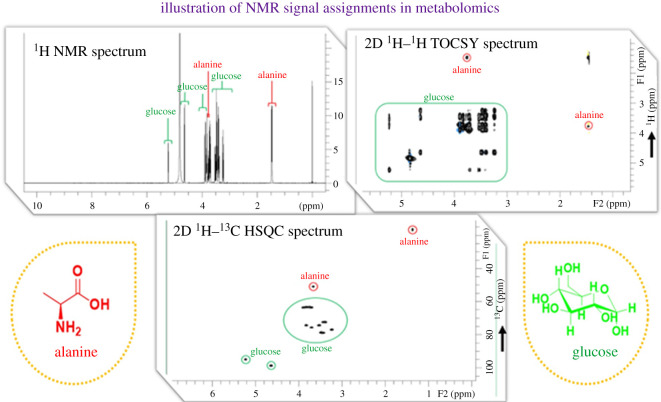
Illustration of assignment of peaks and identification of metabolites from NMR. The 1D and 2D NMR spectra recorded on a mixture of glucose and alanine sample is shown.

The third step is the analysis of NMR spectra, which involves the identification/assignment of metabolites and their quantification using 1D and 2D NMR. The 2D NMR techniques are preferred for obtaining assignments (identification) of the metabolites observed in the NMR spectrum and generally, it is acquired in the beginning phase of the study. The most popular 2D NMR techniques are ^1^H–^1^H total correlation spectroscopy (TOCSY) and ^1^H–^13^C heteronuclear single quantum coherence spectroscopy (HSQC) [36,55]. Figure 2 illustrates the three types of NMR spectra required for peak assignments (1D and 2D NMR). The spectra show the signatures of a mixture of glucose and alanine prepared in an aqueous solvent. Peak assignments are highlighted. The identification of metabolites by NMR is accomplished by the comparison of observed spectral parameters such as the observed chemical shifts, peak intensities and/or coupling patterns with those observed in the reference data of the molecules available in various databases. Ideally, these reference spectra should be available for all measured nuclei (^1^H, ^13^C, ^15^N and ^31^P), and for all types of NMR spectra and for all detected metabolites (about 1000 compounds), at the commonly used spectrometer frequencies (500–800 MHz) [35]. Through collaborative efforts of several metabolomics-based laboratories from around the world, these data are now available in several high-quality, web-based NMR spectral databases containing reference NMR spectra for hundreds of metabolites collected over a wide range of spectrometer frequencies and for a diverse range of nuclei. Most of these databases are freely accessible, including the Human Metabolome Database (HMDB), the Biological Magnetic Resonance Data Bank (BMRB), the Madison-Qingdao Metabolomics Consortium Database (MMCD), the NMRShiftDB2 database and the AIST spectral database in Japan [56–60].

Software such as Chenomx NMRSuite [35] can be used for identifying compounds based on the 1D NMR spectrum. Some open-source programs have been designed to identify compounds by deconvoluting 1D ^1^H NMR spectra [61,62]. Several programs such as complex mixture analysis by NMR (COLMAR), MetaboMiner, PROMEB and ChemSMP are also available to identify compounds from 2D NMR spectra [63–66].

The assignment process is hindered if the spectrum is crowded with overlapping cross-peaks. The conventional approach is to use higher dimensional (2D) experiments. However, higher dimensional experiments require prohibitively long experimental acquisition time (in the order of days). Fast NMR data acquisition is, therefore, important in metabolomics in general. Towards this end, several methods have been developed to shorten the data collection time [67–76]. Our group has developed methods for rapid NMR data acquisition and assignments of molecules in molecules mixtures [65,66,72,73,75,77–79]. These methods provide time gain of a significant magnitude when compared with conventional NMR methods.

## 3. NMR spectroscopy in assisted reproduction

NMR spectroscopy has contributed significantly to identify and quantify biomarkers in the field of ART during the last 3 decades. As discussed above, in NMR, the level of metabolites in each ART sample type can be quantified accurately. Any statistical change (increase or decrease) in the metabolite levels, when compared with an appropriate control, helps to identify specific molecules or biomarkers that are responsible for the condition being studied. However, in a clinical scenario, NMR-based evaluation of various samples from ART is consumptive as NMR tested samples like spermatozoa, testicular tissues, embryos, etc. cannot be used in ART procedures.

**Figure 3.**
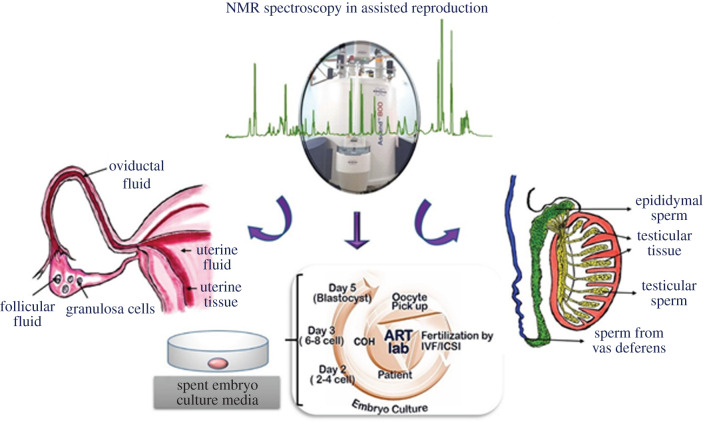
Schematic of the utility of NMR spectroscopy in assisted reproduction and a variety of sample types that have been studied using NMR spectroscopy.

Initial studies on oocytes, spermatozoa and embryos using NMR spectroscopy focused on using ^31^P NMR to understand energy metabolism and related physiological conditions like intracellular pH, motility and growth [80–83]. The application of NMR spectroscopy in assisted reproduction on a variety of experimental samples collected during various stages of ART procedures is schematically represented in figure 3. All the samples are prepared using different methods, and relevant preparation methods are explained below. Following this, the NMR-based studies that have been carried out till date are discussed for each sample type.

### 3.1. Studies on the embryo spent culture medium

#### 3.1.1. Composition of the different embryo culture media

**Figure 4.**
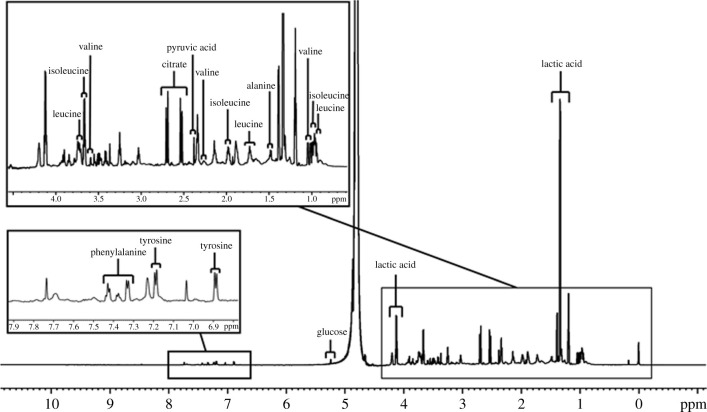
One-dimensional proton NMR spectrum of a one-step embryo culture medium (Vitromed) with peak assignments shown for metabolites.

In clinical IVF, single embryo transfer is gaining popularity to prevent multiple gestations. The blastocyst is preferred over cleavage stage embryos due to a higher implantation rate of single blastocyst transfer [84,85]. A widely used approach is to investigate the metabolome of the culture media in which the embryo is grown, termed as the embryo spent culture media (SCM) as it is a clear reflection of an embryo's physiological or pathological status. Embryo SCM is altered by the developing embryo. A range of metabolic parameters of embryo SCM has been studied using a variety of non-invasive techniques [86–88]. Studies have focused on addressing the association between amino acid uptake, embryo viability and ART outcome [89–91]. NMR can detect the metabolic changes non-invasively and correlate with the embryo growth or implantation [23,92,93]. The preparation of SCM for NMR studies has been described earlier [23]. A representative 1D NMR spectrum of an embryo culture medium is shown in figure 4. The identity/assignment of metabolites is indicated on the peaks.

Human embryo culture was first introduced using a physiological salt solution containing glucose, lactate and pyruvate, supplemented with the patient's serum [94]. Later, the addition of amino acids to the culture medium both in humans and animals demonstrated a positive impact on embryo development and viability [95]. The quantitative analysis was done by Houghton [96] to estimate the amino acid turnover of individual human embryos during the early period of embryo development. Presently, most of the human embryo culture media are enriched with amino acids, energy substrates, growth factors and vitamins.

Currently, two types of media are used in human ART, one-step/single step (monoculture), and sequential culture systems [97]. One-step systems use a single medium component to support the zygote up to the blastocyst stage development. On the other hand, sequential media are used in two stages, first from zygote to the compaction stage and second from the compaction stage to the blastocyst stage. Various factors such as type of incubator, temperature, humidity and air quality result in some changes in the culture medium [98], and collectively, these alterations are known to affect (epi) genetic integrity of the embryo [99]. It has been shown that Sequential ISM1 (ORIGIO) medium resulted in a better embryo quality than that of a one-step Universal IVF (ORIGIO) medium [100]. Another study compared four one-step culture media compositions that varied notably in pyruvate, lactate and amino acids and concluded that blastocyst development was affected by culture media and its interaction with reduced (5%) and ambient (20%) oxygen [101].

Tarahomi *et al.* [101] compared 15 commercial embryo culture media to understand the composition and stability during storage. This study revealed that no two-culture media had an identical composition. Further, significant variation in the stability of the components is observed [102]. These observations suggest that there is no consensus on the media composition for human embryo development. The variation in media components is the primary cause of the conflicting observations in NMR studies on SCM. Sunde *et al*. [98] communicated a strong case for demanding full transparency and the scientific rationale concerning the compositions of embryo culture media as the composition can have a significant influence on the metabolic activity of the embryo.

Initially, SCM metabolites were studied by the modified ultra-microfluorescence method [18]. The global metabolomics approach using NIR and Raman spectra was used for the first time to assess the human embryo viability which could differentiate the embryos that resulted in successful or failed implantation [19]. Subsequently, NMR spectroscopy was applied to profile the human embryo SCM metabolites which revealed a high level of glutamate and alanine/lactate ratio in the embryos that were implanted and delivered successfully [22]. An attempt was made to combine non-invasive metabolomics and chemometric approach for the selection of best quality ART-derived embryos [103]. Subsequently, several groups have used NMR for profiling the metabolites in the SCM. The outcome of such studies is summarized in table 1 and discussed in detail below.

**Table 1.** NMR studies of SCM of human embryos. LAH, laser-assisted hatching; Glc, glucose; Ala, alanine; Lac, lactate; Ile, isoleucine; Gly, glycine; Cit, citrate; Acac, acetoacetate; Thy, thymine; OAc, acetate; Trp, tryptophan; Lys, lysine; Pro, proline; Glu, glutamate; n.a., not available.

study type		study	study population		outcome parameters	metabolic changes observed	
embryos transferred	day, post-fertilization		patients (*N*)	spent media (*N*)		upregulated	downregulated
multiple	3	Seli *et al.* [22]	18	34	implantation and pregnancy in ICSI patients	Glu and Ala/Lac	n.a.
multiple	3	Marhuenda-Egea *et al.* [103]	23	46	implantation and pregnancy for IVF patients	triglycerides, cholesterol compounds and phospholipids were compared	
single and multiple	3	Rinaudo *et al.* [104]	108	228	implantation, ICSI patients	NMR profiling of SCM cannot predict implantation	
single	3	Sánchez-Ribas *et al.* [105]	85	171	trisomy 21 versus monosomy 21 and euploid	caproate and androsterone sulfate	Ile
multiple	2 and 3	Pudakalakatti *et al.* [23]	48	127	implantation for ICSI patients	n.a.	Pyr/Ala
single	3 and 4	Nadal *et al.* [106]	12	39	embryo developmental arrest	(model with poor predictability) NMR profiles alone were not predictive of developmental arrest	
single, double and triple	2	Wallace *et al.* [107]	37	58	IVF/ICSI pregnancy outcome	3-formate/Gly, formate/3-aminoisobutyrate, formate/Ac, formate/Glu and formate/Try	Cit/Ala, aminoisobutyrate/AcAc, 3-aminoisobutyrate/Ala, Cit/formate
single	3 and 5	Kirkegaard *et al.* [84]	161	278	pregnancy, IVF/ICSI patients, positively selected by good prognosis factors	NMR profiles were not predictive of pregnancy	
multiple	3	Uppangala *et al.* [108]	25	50	influence of LAH on embryo developmental potential	Glc, Pyr, Lac Ala, Val, Lys, Pro, Ile, and they were comparable between the groups	
multiple	3	Uppangala *et al.* [109]	34	4	sperm-mediated influence on the embryo developmental potential	Pyr and Pyr/Ala	Ala and Glu

Pudakalakatti *et al.* [23] showed that an embryo with high-implantation potential consumed more pyruvate from the media for their energy requirements, converting it to into lactate by lactate dehydrogenase or into alanine by transamination reaction to avoid the accumulation of ammonia, which causes cell toxicity. The excess alanine thus generated resulted in the increase in alanine in the spent media of embryos with high-implantation potential. Hence, low pyruvate to alanine ratio in the SCM was proposed as a potential biomarker for the selection of embryo with high-implantation potential [23]. On the other hand, a study conducted by Nadal-Desbarats *et al.* [107] characterized the metabolites from individually cultured human embryos on days 3 and 4 [106]. However, this study failed to demonstrate any statistical significance between successful and unsuccessful implantation groups. Wallace *et al.* characterized the SCM metabolites from patients undergoing single, double and triple embryo transfers to predict the pregnancy outcome. This study identified a wide variety of ratios, among which formate/glycine ratio was 17-fold high in day 2 embryo resulting in successful implantation, whereas the citrate/alanine ratio was 22-fold less in the same group. Further, a significant increase in the ratios of formate/3-amino butyrate, formate/acetate, formate/glutamate and formate/tryptophan were characterized for embryos that resulted in successful implantation [107].

While the above studies found the value of NMR analysis in predicting ART outcome based on the level of the metabolites in SCM, Rinaudo *et al.* [104] reported that NMR-based metabolomics profiling cannot be used as a tool for the prediction of embryo implantation potential. Similarly, Kirkegaard *et al.* [84] found no correlation between the NMR metabolic profile of SCM and pregnancy outcome, patient or treatment characteristics.

Notably, the NMR-based profiling of metabolites from SCM has also been used for the differentiation of euploid and aneuploid embryos [105]. Sánchez-Ribas *et al.* [105] investigated the metabolomic signatures of trisomy 21 (T21)/monosomy 21 (M21) and the euploid embryos using NMR and were able to differentiate between normal and aneuploid embryos; they also suggested that isoleucine levels could be used as a differentiator between T21/M21 and normal embryos. Similarly, embryo derived from sperm carrying high DNA damage had reduced pyruvate uptake from the media, reduced alanine released into the media and glutamine intensity was significantly low in the SCM [109]. These approaches suggest the possibilities of using SCM metabolites as non-invasive biomarkers in screening out the genetically abnormal embryos. However, there needs to be more research to understand the association between the turnover of metabolites and the type of genetic lesions and the extent of genetic abnormalities in the embryos.

Laser-assisted hatching (LAH) is one of the commonly used techniques in ART to enhance the implantation process. Since hyperthermia induced by the laser can negatively affect the embryo viability, Uppangala *et al.* [108] conducted a study to understand the impact of LAH on embryo quality. LAH was done on day 2 of the embryonic development and SCM collected 24 h later was subjected to NMR analysis. Metabolites were compared between LAH embryos and the sibling unhatched embryos and results suggested that the LAH does not affect the metabolism of embryos [108].

The metabolic profiling of SCM has also been carried out in mouse and bovine embryo culture systems. Unlike human embryos, murine models allow the selective manipulation of embryo growth conditions and to monitor the effectiveness of such changes on the embryo metabolism. Historically, bovine embryos have been used to study early embryo developmental stages as the early developmental mechanism in cattle and humans are very similar. Such studies have always contributed towards the advancement in human IVF procedures. The details of these studies are summarized in table 2.

**Table 2.** NMR studies of animal embryo SCM. CL, cleavage stage; BL, blastocyst stage; IVF, *in vitro* fertilization; PA, parthenogenetically activated; Cit, citrate; Pyr, pyruvate; Lys, lysine; Ac, acetate; Phe, phenylalanine; His, histidine; Trp, tryptophan; Val, valine; Glc, glucose; Lac, lactate; Thy, thymine; Pro, proline; Ala, alanine, Ile, isoleucine; n.a., not available.

study model	study type	study	study population	analysis carried out	metabolic changes observed	
					upregulated	downregulated
bovine	2-day culture, IVF-derived and parthenogenetic-activated embryos	Rubessa *et al.* [110]	CL 20, BL 20,	blastocyst development	Cit, Pyr and Lys	Myo-inositol
			IVF 20, PA 20	IVF versus PA	Phe	↓OAc
	individual culture and group culture (30/drop)	Perkel *et al.* [111]	113	fast-growing and slow-growing embryos	4 cell-Pyr	4 cell: n.a.
					8 cell and 16 cell: His, Trp, Val	8 cell and 16 cell: n.a.
					2 cell and 16 cell: Ile, Leu	2 cell and 16 cell: n.a.
	group culture system	Rubessa *et al.* [83]	male 20, female 20	sex determination of embryo at early (1–3 days) and late development stage (5–7 days)	1–2 days: Val	1–2 days: n.a.
					5–7 days: n.a.	5–7 days: Pyr
mouse	group culture (10/drop)	D'Souza *et al.* [112]	185	embryo carrying induced DNA lesion on day 3.5	Glc, Lac, Pyr, Thy, Pro, Val, Ala, Ile and Lys	n.a.
	individual culture	D'Souza *et al.* [93]	118	blastocyst *in vitro* outgrowth	Pyr and Lac	Pyr/Ala
	individual culture	D'Souza *et al.* [113]	90	blastocyst	Pyr	Ala, Pyr/Ala and Pyr/Lac

Perkel & Madan [111] carried out the first NMR-based metabolomics study of individually cultured bovine embryos. The study revealed a difference in the requirement of metabolites between slow-growing (SG) and fast-growing (FG) bovine embryos [111]. Global metabolome analysis of spent culture medium can also be used for gender differentiation [83]. A study conducted by Rubessa *et al.* [83] in bovine embryos showed that gender-dependent metabolite differences can be seen in the global metabolome analysis. While the bovine model is extensively used to understand the pathophysiology of human reproductive function, cattle are known ruminants; hence, one can expect significant differences in the metabolism between bovine and humans. Since the present review is focused on biomarkers for human IVF, there is a potential pitfall in extrapolating bovine data to the clinical IVF.

D'Souza *et al.* [93] used mouse embryo SCM to predict the implantation ability of the embryos which showed the increased requirement of pyruvate and lactate accompanied by a significant reduction of pyruvate/alanine ratio in the SCM. Further, sperm-induced genetic lesions in the embryos are associated with a significant increase in the uptake of pyruvate and reduced pyruvate/alanine ratio in SCM [113]. Data from animal studies provide only fundamental knowledge but cannot be directly extrapolated to human ART due to species-specific differences in the metabolic requirements, chromatin architecture and developmental timelines.

Despite the promising observations by several researchers, embryo SCM metabolomics by NMR has not been implemented clinically, possibly due to technical limitations in establishing collaboration between ART clinics and NMR facilities, the time requirement for NMR analysis and the need of experts to interpret the NMR results. Further, the discrepancies observed so far could be attributed to the great heterogeneity in the variables used in each study, such as the composition of embryo culture media, culture conditions, collection technique, handling, and transportation of SCM, and measurement technique. Thus, the standardization of variables is essential before using NMR metabolomics as a complementary or independent tool for embryo selection in human ART.

### 3.2. NMR studies of follicular fluid

The accomplishment of oocyte developmental competence is crucial for the formation of viable, good quality embryos. The oocyte growth and development depend primarily on the nurturing microenvironment of the follicle, including the FF. The FF consists of exudates of the circulating plasma, secretion from the follicular and granulosa cells and essential substances like growth factors, cytokines, amino acid, energy substrates, steroids, lipids and cholesterol that are necessary for the oocyte maturation and fertilization *in vivo* [114,115]. Several NMR studies have been performed in search of a biomarker from FF for assessing the embryo quality (table 3). The preparation of FF for NMR analysis has been described [118].

**Table 3.** NMR studies of the follicular fluid. Lac, lactate; Glc, glucose; HDL, high-density lipoprotein; Pro, proline; Leu, leucine; Ile, isoleucine; Chol, choline; ChoP, phosphocholine; GPC, glycerophosphocoline; Glyc, glycerol; TMA, trimethylamine; Pyr, pyruvate; Ac, acetate; Gly, glycine; Gln, glutamine; Ala, alanine; Asp, aspartate; Asn, asparagine; Cit, citrate; Cr, creatinine; 3-HB, 3-hydroxybutyrate; Lys, lysine; Met, methionine; Phe, phenylalanine; n.a., not available; FF, follicular fluid.

study model	study type	study	study population			metabolic changes observed	
			no. of patients	no. of FF	analysis carried out	upregulated	downregulated
human	oocyte donors women undergoing IVF	Piñero *et al.* [114] Wallace *et al.* [116]	30	30	oocyte quality	42 metabolites identified	
			58	108	developmental and implantation potential of the embryo	2 cell: Lac and Chol/ ChoP	2 cell: Glc and HDL
						implantation: Pro, Lac and Leu+ Ile	implantation: Glc
	women undergoing natural cycle versus IVF/ICSI	McRae *et al.* [117]	10	10	effect of exogenous hCG administration during pre- and mid-ovulatory period	FF: Lac, Pyr	FF: Glc
						plasma: Glc and Ac	plasma: glycoprotein, Gly and trimethylamine
	PCOS patients	Zhang *et al.* [118]	51	51	metabolic profile and oocyte quality	glycoprotein, Ac and cholesterol	Lac, Gln, Pyr and Ala
	PCOS patients	Nunzia *et al.* [119]	41	41	AMH versus FF metabolites	Glc	Lac, Pyr and Ala
	different stages of endometriosis	Marianna *et al.* [120]	30	60	metabolic profile and oocyte quality	Lac	Lys, Asp, Chol, Pro, Ala, Leu, Val and ChoP
	benign and malignant	Morelli *et al.* [121]	20	20	metabolic profile and fertility preservation	Cit, Cr, Glyc, GPC and Glc	Asn, Asp, Pro, cholesterol, Chol, Lac and lipids
	endometriosis	Karaer *et al.* [122]	24	24	metabolic profile	Lac, β-Glc, Pyr and Val	n.a.
	different female factor infertility	Morelli *et al.* [123]	53	53	metabolic profile	PCOS: Glc, Glycerol, Cr	Ac, Leu, Thr, β-HB
						endometriosis: Glc, Lac	Ac, β-HB, Cit and Val
sheep, pig and cow	FF, ovarian venous blood	Gosden *et al.* [124]	sheep 3, pigs 5, cows 4		metabolic profile	Ac, Ala, Cr/Creatine, Gly, D-3-HB, Lac and Val identified	
mare	FF and serum	Gérard *et al.* [125]	FF 20 and serum 20		early, late and pre-ovulatory stage dormant follicle	Ala and lipoprotein	TMA, glyco-conjugates, Ac and Glc
pig	FF	Bertoldo *et al.* [126]	summer small 8 and large 15, winter small 9 and large 15		metabolic profile of good and poor follicular environments	follicular size alone: Glc	follicular size alone: Lac, hypoxythine, Ac, inositol, TMA, Ala, Leu, Lys, Met and Phe
						follicular size and season: Glu, Gly, *N*-acetyl group and uridine identified	
						season alone: succinate	n.a.
mare, sow and cow	FF	Gérard *et al.* [115]	small 5 and large 5 per animal group		metabolic profile of small and large follicle from three species	α and β Glc	n.a.

In general, the NMR-based metabolomics characterization of FF reveal variations in the composition in relation to species [124], oestrous cycle stage [117,125], seasonal influence [126], underlying pathology such as PCOS [118,119,123], endometriosis [120,122,123], benign and malignant ovarian cancers [114], other infertility conditions [123] and the size and quality of the developing oocyte [114,115,126]. Association has also been established between FF composition and the reproductive potential of the developing oocyte and embryo [116]. Gosden *et al.* [124] carried out the first NMR-based study to characterize the FF metabolites from cow, sheep and pig and showed a species-specific difference in the composition of metabolites.

Few studies have investigated the clinical value of FF analysis by NMR in human ART. Wallace *et al.* [116] for the very first time studied FF samples from women undergoing IVF and observed a high level of lactate and choline/phosphocholine, and a low level of glucose and high-density lipoprotein (HDL) in the FF collected from the oocyte that fertilized and formed good embryos, compared with the FF from the oocyte which fertilized but failed to develop further. Similarly, metabolomic profiling of FF was found useful in predicting the developmental competence of the human oocyte, thereby facilitating embryo selection [117].

Factors such as the day of follicular aspiration, size of the follicle, synchrony of the follicular cohort within the ovary and patients' pathology do influence the follicular microenvironment. Gonadotrophin stimulation in ART normally results in the recruitment of multiple follicles where time-dependent increase in the follicle size and their synchronous development play a crucial role in producing mature and competent oocytes. Follicle size-dependent variation in metabolic profile was observed in the FF composition [115]. Gérard *et al*. studied the FF of the small and large follicles collected from three different species; cow, sow and mare. Even though there exists a species-specific difference in the FF composition, in general, all species were found to have a higher concentration of α and β glucose in the large follicles (possibly mature) compared with the small follicles (possibly immature) [115]. However, it has been reported that not only the follicle size but also the season and the quality of the follicle have an influence on the porcine FF composition [126]. Nonetheless, at this juncture, there is no conclusive evidence to apply FF metabolites as biomarkers in human ART.

PCOS is one of the major causes of female factor infertility, affecting 5–10% of women of reproductive age [123]. The major metabolic dysregulation among PCOS patients is glucose intolerance due to insulin resistance, dyslipidaemia and hyper-androgenization which eventually influences the follicular environment and oocyte quality. Biochemical studies of the FF found that carbohydrate/glucose metabolism is impaired due to hyperinsulinemia in PCOS patients, whereas NMR-based metabolic profiles have suggested that glucose is positively correlated with the serum level of anti-Müllerian hormone (AMH) which is a marker for ovarian reserve. On the other hand, the level of lactate, pyruvate and alanine were negatively correlated with AMH. From the metabolomics point of view, glucose, lactate and pyruvate are directly involved in the glycolytic pathway and cumulus and granulosa cells that are responsible for the transfer of energy intermediate of the glycolytic pathway to the developing oocyte are unable to do so due to the insulin resistance, leading to an elevated level of glucose and overuse of the alternative pathways [119]. A significant difference in the FF metabolites between PCOS and non-PCOS patients and their impact on oocyte quality and fertilization outcome was demonstrated by NMR [123]. Hence, the alterations in pyruvate metabolism, glycolysis, and amino acid metabolism in FF help in explaining the PCOS pathogenesis and its impact on the oocyte environment within the follicle [118].

Endometriosis is another major cause of female infertility which compromises the oocyte quality and implantation potential. The metabolomics-based analysis of FF collected from the patient with endometriosis showed elevated concentration of glucose, lactate and pyruvate indicating more demand for glycolysis and anaerobic metabolism compared with the healthy control [120,122,123]. On the other hand, the concentration of acetate, citrate and β-hydroxybutyrate were reduced in the FF of endometriosis patients [123]. Valine is an essential branched chain amino acid attributed to the inflammatory response in endometriosis patients. Studies conducted by Castiglione Morelli *et al.* [123] and Marianna *et al.* [120] reported a low level of FF valine, whereas Karaer *et al.* [122] showed an elevated level in the endometriosis patients compared with the healthy control. Further, the concentration of few fatty acids, lysine, choline, glucose, aspartate, alanine, leucine, valine, proline, phosphocholine, total LDH as well its LDH-3 isoform were found to be reduced in endometriosis patients compared with the healthy control [120].

These observations can aid-in understanding the impact of specific pathological conditions such as PCOS and endometriosis on the follicular microenvironment and how altered metabolites can further affect the functional ability of the oocytes in ART.

Restoration of fertility in cancer survivors is a challenging task and still experimental. It is well known that cancer affects several metabolic pathways in general. Castiglione Morelli *et al*. [121] looked at the metabolite profile of FF from cancer patients which showed a reduction in the concentration of asparagine, aspartate, proline, cholesterol, choline, lactate, lipids compared with the healthy group. Also, an upregulation of the citrate, creatine, glycerol, glycerophosphocholine and glucose concentration was observed in cancer patients compared with the healthy control [121]. Hence, NMR-based metabolomics could serve as a prognostic tool for identifying and selecting the oocytes in relation to FF metabolites, and thereby help in predicting the ART outcome when cancer survivors are undergoing fertility restoration treatment.

### 3.3. NMR studies of blood serum/plasma

Examination of plasma composition during the follicular and periovulatory phases of the menstrual cycle in patients undergoing fertility treatment provides insights into the underlying pathology. PCOS is known to impair oocyte quality, endometrial receptivity and embryo implantation potential, which can be captured through metabolites from several body fluids including blood serum or plasma. Standard protocols are followed for the collection and preparation of the serum or plasma samples used in NMR [29,30]. A study conducted by Zhao *et al.* showed that the difference in serum metabolomic profile in PCOS and control patients was characterized by elevated glycolysis, reduced tricarboxylic acid cycle (TAC), reduced ratio of branched chain amino acid/aromatic amino acid in PCOS. The results also suggested upregulated concentration of serine, threonine, phenylalanine, tyrosine and ornithine in PCOS patients could be the main reason for ovulatory dysfunction [29].

RoyChoudhury *et al.* explored the value of serum metabolites to understand the pathophysiology of recurrent implantation failures. The concentration of eight metabolites (valine, adipic acid, lysine, creatine, ornithine, glycerol, d-glucose, urea) were elevated in the serum of patients with recurrent implantation failure compared with control [127]. Details of these studies are stated in table 4.

**Table 4.** NMR studies of the human blood serum/plasma. Ser, serine; Thr, threonine; Phe, phenylalanine; Val, valine; Leu, leucine; Gly, glycine; Orn, ornithine; Glc, glucose; Lys, lysine; n.a., not available.

study type	study	*n*	analysis carried out	metabolic changes observed	
				upregulated	downregulated
PCOS	Zhao *et al.* [29]	265	potential metabolic profiles for different phenotypes of PCOS	Ser, Thr, Phe, Tyr, Val, Leu and Orn	Gly
repeated implantation failures (RIF) versus repeated implantation success (RIS)	RoyChoudhury *et al.* [127]	52	metabolic dysfunction associated with RIF	Vali, adipic acid, Lys, creatine, Orn, glycerol, d-Glc and urea	n.a.

These studies have significance not only in identifying novel metabolic biomarkers that predict specific pathological conditions but also in better understanding of the metabolic pathways directly affected during these diseases, which may provide a rationale for the development of novel therapeutics.

### 3.4. NMR studies of oocytes and embryos

The oocyte contributes 70–80% towards the successful fertilization and further embryonic development. Hence, knowing the biochemical status of the oocyte helps to enhance the reproductive outcome. One of the rate-limiting steps in ART is the selection of a single embryo with the highest implantation potential. Understanding the embryo kinetics and metabolism by NMR may help to tackle this problem. Towards this end, a few studies have been performed, the details of which are summarized in table 5. However, it is important to note that in-cell analysis of oocytes/embryos is restricted to non-human oocytes and embryos due to ethical restriction in exposing them to invasive preparation such as centrifugation and NMR as described earlier [129]. Rubessa *et al.* [110] used NMR to study the metabolic requirements of bovine gametes and embryos during the *in vitro* production and thereby made modification in the culture media composition. This study analysed the pyruvate, lactate and alanine concentration of SCM during of gametes co-incubation. The concentration of pyruvate was significantly reduced during the culture of oocyte–cumulus complex (OCC) alone and OCC co-incubated with sperm. This observation helped investigators to modify the embryo culture media composition by reducing the concentration of pyruvate and lactate which improved the bovine embryonic development [110].

**Table 5.** NMR studies of the oocytes and embryos. Pyr, pyruvate; Lac, lactate; n.a., not available.

study type	study	study population		analysis carried out	metabolic changes observed	
		no. of animals	*n*		upregulated	downregulated
bovine oocyte	Rubessa *et al.* [110]	n.a. (911 oocytes)	35+	evaluation of energy consumption during gamete co-incubation and reformulation of IVF media	n.a.	Pyr and Lac
mouse embryo	Lyman *et al.* [128]	n.a.	402	embryo viability in real time using NMR	n.a.	n.a.

In-cell NMR analysis of the mouse embryos was conducted to understand the embryo viability and the study found that in-cell NMR analysis has an adverse effect on the *in vitro* development of the mouse embryos. However, the observed effect was mainly because of the medium used for the analysis. Hence, modification in the analysing medium may provide better information on embryo viability [128]. However, it is important to note that exposing embryos to NMR may have potential long-term detrimental effects, hence practically not possible to apply this method in a clinical setting.

### 3.5. NMR studies of testicular tissue

The biochemical status of the testicular tissue has a direct influence on the process of spermatogenesis. NMR-based metabolomics helps to understand the hidden cause of male infertility. In the experimental set-up, making good use of NMR spectroscopy, characterization of metabolites could help in understanding bio-dysfunction of gonads and aid in developing therapeutic options [130]. The details of such studies are summarized in table 6. Sample preparation for NMR analysis has been described earlier [133]. Human testis was initially studied by Chew *et al.* [131] using ^31^P NMR and found that that phosphomonoester (PM)/β adenosine triphosphate (ATP) and PM/phospho-diester ratios were low in infertile patients compared with the healthy control, whereas the ratio of phosphate/PM was high in patients with primary testicular failure and chronic tubular obstruction compared with the control. Rat testicular tissue had a high concentration of lactate, alanine, phosphatidylcholine and creatinine, which help to meet the energy requirements and to maintain the cell viability [132]. Jarak *et al.* [133] addressed the association between rat testicular senescence and the related decline of fertility using ^31^P NMR which revealed an age-dependent decline in the antioxidant metabolites like betaine, creatine and glutathione; the elevated level of phenylalanine and tyrosine; decrease in nucleotide synthesis (IMP, CMP, ATP) and increase in testicular content of phospholipid (choline, ethanolamine, myo-inositol, glycerol) precursors. At present, results are inconclusive to suggest the utility of testicular tissue NMR analysis in the diagnosis and clinical management of infertility.

**Table 6.** NMR studies of the testicular tissue. PM, phosphomonoester; ATP, adenosine triphosphate; Pi, inorganic phosphate; n.a., not available; Cr, creatine; Lac, lactate; Ala, alanine; Phe, phenylalanine; Tyr, tyrosine.

model	study	study population		analysis carried out	metabolic changes observed	
		patients/animals	samples		upregulated	downregulated
human	Chew *et al.* [131]	23	23	testicular metabolic integrity and differentiation of normal testicles	Pi/PM	PM/β-ATP and PM/phospho-diester
rat	Griffin *et al.* [132]	6	1	differential metabolomics profiling	Cr, phosphatidylcholine, Lac and Ala	n.a.
	Jarak *et al.* [133]	40	5	senescence and declining reproductive potential	choline, ethanolamine, myo-inositol, glycerol, Phe and Tyr	betaine, Cr and glutathione

### 3.6. NMR studies of spermatozoa

The structural and functional integrity of spermatozoan can seriously affect its fertilizing ability and the developmental competence of embryos. NMR has been used extensively to address the biochemical environment of spermatozoa (table 7). ^31^P NMR analysis of male gametes in the sea urchin, turbot, *Xenopus laevis*, etc. have addressed the structure and differences in functional characteristics [80,139–142]. Using NMR, Smith *et al.* [143] found that bovine spermatozoa use an unknown energy source for the phosphorylation of ATP and thereby maintains acidic cytosolic pH. This study also found that prolonged incubation of sperm with media promotes the accumulation of lactate in the surrounding media and leads to an exchange of internal K^+^ for H^+^, thereby reducing the intracellular sperm pH.

**Table 7.** NMR studies of the spermatozoa. Lac, lactate; GPC, glycerophosphorylcholine; Chol, choline; n.a., not available.

model	study	study population		analysis carried out	metabolic changes observed	
		animals/subjects	samples		upregulated	downregulated
rhesus macaque	Lin *et al.* [134]	6	n.a.	season-dependent energy metabolism	formate, carnitine and acetyl carnitine	Lac
human	Paiva *et al.* [135]	9	3	endogenous metabolism	42 metabolites identified	
	Reynolds *et al.* [136]	n.a.	n.a.	progressive motility versus oxidative phosphorylation and lactate fermentation	Lac and bicarbonates	n.a.
	Reynolds *et al.* [137]	n.a.	n.a.	metabolomics of live sperm (40% and 80%)	Lac, lipid and Chol/GPC	n.a.
	Calvert *et al.* [138]	97	n.a.	glycolysis and oxidative phosphorylation for viable sperm population	Lac	n.a.

NMR studies have proved useful for the development of metabolomic markers that signal sperm metabolic impairments. The preparation of spermatozoa for ^1^H NMR has been described earlier [135]. NMR profiling of monkey spermatozoa collected during different seasons (i.e. autumn and spring) have shown that there exists a seasonal-dependent variation in the metabolic profile characterized by a high level of formate in the autumn season and high level of carnitine and acetylcarnitine during the spring season [134]. This study also found that glycolysis plays a major role to yield ATP in the monkey's sperm. Extensive intracellular metabolomics characterization of 69 intracellular metabolites in human spermatozoa done by the combined application of ^1^H NMR spectroscopy and gas chromatography–mass spectrometry (GC-MS) [135] may describe their function in male gamete physiology and to help in exploring potential causes for sperm dysfunction.

Reynolds *et al.* profiled percoll gradient separated live human spermatozoa to demonstrate differential metabolomic signature in relatively low concentration of sperm (approx. 3 × 10^6^ ml^−1^) which could open up the possibilities of developing metabolomics-based diagnostics to test poor quality ejaculates [137]. The same group used dissolution dynamic nuclear polarization (dDNP) to enhance NMR sensitivity to study the correlation between the energy metabolism and sperm motility in human which showed ^13^C labelled pyruvate was converted significantly into lactate and bicarbonate, indicating the active glycolytic and oxidative phosphorylation in progressively motile spermatozoa [136].

### 3.7. NMR studies of seminal fluid/plasma

Seminal fluid has important roles in sperm survival and overall fertilization success. Biochemical changes in seminal plasma composition may alter the fertility potential of the spermatozoa. It is, therefore, important to consider the implication of different biomarkers in seminal plasma, both in the diagnosis and treatment of male infertility. Studies conducted on seminal plasma using NMR are summarized in table 8. Seminal plasma preparation for NMR analysis has been described earlier [86]. The metabolome of human seminal plasma secretion in patients with vasal aplasia and non-obstructive infertility revealed that the ratio of citrate/choline and spermine/choline were twofold higher in these patients compared with fertile subjects [144]. Further, an attempt to use NMR profiling of seminal plasma to distinguish the obstructive and non-obstructive forms of azoospermia and different forms of spermatogenic failure has shown that glycerophosphorylcholine (GPC)/choline ratio can serve as an important parameter to differentiate between different forms of spermatogenic failure [145].

**Table 8.** NMR studies of the seminal fluid/plasma. GPC, glycerophosphorylcholine; Cit, citrate; Lac, lactate; Chol, choline; Glc, glucose; Cit, citrate; Ala, alanine; His, histidine; GPE, glycerylphosphorylethanolamine; UMP, uridine 5 monophosphate; Thy, thyrosine; Phe, phenylalanine; Lys, lysine; Fra, fructose; Trp, tryptophan; Cr, creatinine; PCr, creatine phosphate; Gly, glycine; n.a., not available.

model	study	study population		analysis carried out	metabolic changes observed	
		animals/subjects	samples		upregulated	downregulated
human	Lynch *et al*. [144]	19	19	vas deferens obstruction and sperm antibodies	Cit/Chol and spermine/Chol	n.a.
	Hamamah *et al*. [145]	60	60	differential metabolomics profiling of different infertility condition	n.a.	GPC, Cit and Lac
					glycerylphosphorylethanolamine (GPE), Cit/Lac, GPC/Lac and GPE/GPC are significant	
	Tomlins *et al*. [146]	n.a.	n.a.	time-dependent changes	phosphorylcholine to Ch and UMP to uridine identified	
	Sharma *et al*. [147]	37	n.a.	effect of new injectable male contraceptive	n.a.	Glu, Lac, GPC, Chol, Cit/Lac and GPC/Chol
	Gupta *et al*. [148]	230	230	effect of *Mucuna pruriens*	Ala, Cit, GPC and His	Phe
	Gupta *et al*. [149]	185	185	oligoazoospermia	Phe and Tyr	Ala, Cit and GPC
	Gupta *et al*. [150]	230	230	effect of *Withania somnifera*	Lac, Ala, Cit, GPC, His and Glu	Phe
	Bonechi *et al*. [151]			different infertility condition	GPC, Chol, Phe, Cit, Lac, His and uridine identified	
		83	83			
	Zhang *et al*. [152]	63	63	asthenozoospermia	19 metabolites and 5α-cholesterol and 7-ketocholesterol are upregulated	
	Darbandi *et al*. [153]	151	151	ROS induced changes	TMA N-oxide	Trp and Tyr/Tyrosol
	Mumcu *et al*. [154]	59	59	oligoastheno teratozoospermia	Tyr	Cit, spermine, putrescine, α-keto glutaric acid, Val, Lac, Cr, Lys, Arg and Gln
	Mehrparvar *et al*. [155]	29	29	teratozoospermia	Cit, Ch, d-Glc, l-Tyr, l-Ala, l-Leu, l-Pro, l-Lys, myoinsitol, l-Lac, Thr, Pyr, Gln, Val and Ile	l-Glu, taurine and cholesterol
rat	Ebrahimi *et al*. [156]	24	n.a.		Ala, Lac and His	n.a.

Time-dependent biochemical changes were analysed in the seminal fluid by co-incubation with EDTA. NMR profile revealed a rapid observable change in the conversion of phosphorylcholine to choline and uridine-5-monophosphate (UMP) to uridine [146]. This observation may help in understanding the interaction between spermatozoa and seminal plasma and to address abnormalities in the seminal liquefaction process.

Seminal plasma analysis by NMR was used to test the effects of natural products on sperm functional improvement. *Mucuna pruriens* therapy rectifies the hormonic balance of endogenous metabolites such as alanine, citrate, GPC, histidine and phenylalanine content in seminal plasma and improved the semen quality in infertile men [148]. The effectiveness of *Withania somnifera* plant extract in male infertility management showed restoration of lactate, alanine, glutamate, citrate, GPC and histidine in the human seminal plasma over a period of three months and reduced phenylalanine level [150]. Similarly, NMR helped in understating the effects of *Eurycoma longifolia* extracts in improving rat sperm concentration by studying the level of alanine, lactic acid and histidine in the post-treated rat seminal plasma [156].

The use of NMR for metabolic profiling of seminal plasma has rendered it as a diagnostic tool for male infertility. Lower levels of seminal plasma metabolites such as alanine, citrate and GPC and a higher level of tyrosine, and phenylalanine were found in oligozoopsermic (decreased sperm number in the ejaculate) men [149]. Asthenozoospermia (decreased sperm motility) was characterized by the upregulation or downregulation of 19 metabolites responsible for lipid metabolism, phospholipids (choline) metabolism, cholesterol metabolism, nucleoside metabolism, the Krebs cycle and energy metabolism [152]. Further, a high level of 5α-cholesterol and 7-ketocholesterol was clearly evident in asthenozoospermic patients, indicating the degree of oxidative stress in the group compared with the control [152]. The level of citric acid, choline, d-glucose, tyrosine, alanine, proline, leucine, lysine, myo-inositol, lactic acid, threonine, pyruvate, glutamine, valine and isoleucine were high and glutamic acid, cholesterol and taurine levels were low in the seminal plasma of teratozoospermia (increased number of morphologically abnormal spermatozoa) [155]. It has been proposed that NMR signature of seminal plasma along with semen analysis data can be used to categorize the different infertility condition [151]. The model showed a well discrimination between leukocytospermia (pus cells in the ejaculate) and concomitant varicocele/ex varicocele, also testicular cancer, necrozoospermia (increased number of dead spermatozoa) and azoospermia (no sperm in the ejaculate) from other conditions. The metabolites including GPC, a cluster of metabolites consist of choline, phenylalanine, citrate, lactate, and histidine and uridine played an important role in the clustering different infertility condition [151]. Oxidative stress can significantly influence functional and genetic integrity of spermatozoa. Elevated seminal plasma reactive oxygen species (ROS) can result in the downregulation of tyrosine/tyrosol and tryptophan and upregulation of the trimethylamine N-oxide levels [156].

Since seminal plasma is a complex biological fluid, NMR application in studying the metabolites so far have provided general information on the association between type of spermatogonial disorders [144–146,149,151,152,155], and effect of treatment modalities on change in the level of metabolites [148,150,156]. Though these observations may not have any direct implications on human ART, development of specific biomarkers in predicting genetic and functional integrity of male gametes non-invasively may open up a new era in the non-invasive sperm selection.

## 4. Emerging trends and future challenges

Non-invasive evaluation of embryos in ART clinic has advanced by multiple emerging techniques such as NMR, Raman, NIR and MS. Lately, the ability to predict embryo potential has been enhanced by introduction of time-lapse imaging systems (TLS) in ART clinics. It allows the assessment of embryo quality without physically removing them from the incubator. TLS has been used to explore the associations between the morphologic, morphometric and morpho kinetic parameters of oocytes, zygotes and embryos, and their associations with live birth [15,157].

The field of NMR is constantly evolving, and several new techniques are being developed to improve upon the limitations of existing NMR-based metabolomics. These include hyperpolarization methods [158], ultrafast 2D NMR methods [37,55], pure-shift NMR techniques [159,160] and hybrid NMR approaches [24,26,161,162]. Inhomogeneous intact tissue samples can be analysed with the use of high-resolution magic-angle spinning (HRMAS) spectroscopy [163,164]. Using HRMAS, tissue samples can be examined without the need for sample extraction or other sample preparation steps [163,164]. Hyperpolarization methods such as DNP can be used to enhance the NMR signal intensities of metabolites, hence improving the sensitivity of NMR experiments [165]. In principle, the sensitivity enhancement of three orders of magnitude can be obtained [166,167]. DNP allows for the detection of metabolites present in very low concentrations [168].

Together, with the advent of new NMR methods and stronger magnets, NMR spectroscopy is providing a complementary tool to explore various aspects of assisted reproduction. There are several isotope labelling methods in NMR-based metabolomics [78] that can be employed in ART. NMR serves as a research tool to explore and unravel the metabolic signature of embryos and to discover biomarkers. However, in the clinical IVF centre, an NMR set-up is not needed. Instead, a simple assay or kit-based approach can be developed to rapidly detect and quantify the biomarkers on-site that have been discovered by NMR. For detection of the metabolites at the IVF site, two approaches can be implemented: (i) a colorimetric assay involving enzymatic conversion of metabolites followed by detection and quantification using absorption spectroscopy, or (ii) HPLC. Both these methods can be calibrated with known standards and cross-validated with the quantitative results obtained from NMR spectroscopy. Our laboratories are currently working in this direction to develop IVF diagnostic kits.

Precision medicine, an emerging branch of medicine, functions with prospects of the customization of healthcare (i.e. medical practices, decisions and treatments are tailored to individual patients [169]). The introduction of automatized embryo morphological evaluation using time-lapsed imaging in conjunction with using artificial intelligence (AI) is an attractive possibility [15]. Integration of NMR-based metabolomics and clinical parameters into an AI model will have powerful diagnostic and prognostic values in assisted reproduction. This will help embryologists to optimize techniques and personalize the treatment to maximize the reproductive outcome in the field of ART.

## Data accessibility

This article has no additional data.

## References

[1] Sharma R, Biedenharn KR, Fedor JM, Agarwal A 2013 Lifestyle factors and reproductive health: taking control of your fertility. Reprod. Biol. Endocrinol. 11, 66 (10.1186/1477-7827-11-66) 23870423 PMC3717046

[2] Ilacqua A, Izzo G, Emerenziani GP, Baldari C, Aversa A 2018 Lifestyle and fertility: the influence of stress and quality of life on male fertility. Reprod. Biol. Endocrinol. 16, 115 (10.1186/s12958-018-0436-9) 30474562 PMC6260894

[3] Kushnir VA, Barad DH, Albertini DF, Darmon SK, Gleicher N 2017 Systematic review of worldwide trends in assisted reproductive technology 2004–2013. Reprod. Biol. Endocrinol. 15, 6 (10.1186/s12958-016-0225-2) 28069012 PMC5223447

[4] Steptoe PC, Edwards RG 1978 Birth after the reimplantation of a human embryo. Lancet 312, 366 (10.1016/S0140-6736(78)92957-4) 79723

[5] ESHRE Monitoring. 2018 More than 8 million babies born from IVF since the world's first in 1978. See https://www.sciencedaily.com/releases/2018/07/180703084127.htm.

[6] Health D of R. 2019 *Assisted reproductive technology 2017: fertility clinic success rates report*. Atlanta, GA: National Centre for Chronic Disease Prevention and Health Promotion.

[7] Report NS 2018 *Assisted reproductive technology 2016: national summary report*. Atlanta, GA: National Centre for Chronic Disease Prevention and Health Promotion.

[8] Wyns C et al 2020 ART in Europe, 2016: results generated from European registries by ESHRE. Hum. Reprod. Open 3, 1–17. 10.1093/hropen/hoaa032 PMC7394132 32760812

[9] Luke B, Brown MB 2007 Contemporary risks of maternal morbidity and adverse outcomes with increasing maternal age and plurality. Fertil. Steril. 88, 283–293. (10.1016/j.fertnstert.2006.11.008) 17258214 PMC1955760

[10] Tiitinen A 2019 Single embryo transfer: why and how to identify the embryo with the best developmental potential. Best Pract. Res. Clin. Endocrinol. Metab. 33, 77–88. (10.1016/j.beem.2019.04.001) 31005505

[11] Meczekalski B, Szeliga A, Podfigurna A, Miechowicz I, Adashi EY 2020 Assisted reproductive technology outcome in United States of America and Australia with New Zealand: comparison of annual reports 2005–2016. Gynecol. Endocrinol. 36, 1–9. (10.1080/09513590.2020.1737006) 32172637

[12] Cummins JM, Breen TM, Harrison KL, Shaw JM, Wilson LM, Hennessey JF 1986 A formula for scoring human embryo growth rates in in vitro fertilization: its value in predicting pregnancy and in comparison with visual estimates of embryo quality. J. Vitr. Fertil. Embryo Transf. 3, 284–295. (10.1007/BF01133388) 3783014

[13] Rocha JC, Passalia F, Matos FD, Maserati Júnior MP, Alves MF, de Almeida TG, Cardoso BL, Basso AC, Nogueira MFG 2016 Methods for assessing the quality of mammalian embryos: how far we are from the gold standard? JBRA Assist. Reprod. 20, 150–158. (10.5935/1518-0557.20160033) 27584609 PMC5264381

[14] Conaghan J 2014 Time-lapse imaging of preimplantation embryos. Semin. Reprod. Med. 32, 134–140. (10.1055/s-0033-1363555) 24515908

[15] Tran D, Cooke S, Illingworth PJ, Gardner DK 2019 Deep learning as a predictive tool for fetal heart pregnancy following time-lapse incubation and blastocyst transfer. Hum. Reprod. 34, 1011–1018. (10.1093/humrep/dez064) 31111884 PMC6554189

[16] Botros L, Sakkas D, Seli E 2008 Metabolomics and its application for non-invasive embryo assessment in IVF. Mol. Hum. Reprod. 14, 679–690. (10.1093/molehr/gan066) 19129367 PMC2639446

[17] Leese HJ 2012 Metabolism of the preimplantation embryo: 40 years on. Reproduction 143, 417–427. (10.1530/REP-11-0484) 22408180

[18] Leese HJ, Hooper MAK, Edwards RG, Ashwood-Smith MJ 1986 Uptake of pyruvate by early human embryos determined by a non-invasive technique. Hum. Reprod. 1, 181–182. (10.1093/oxfordjournals.humrep.a136376) 3624425

[19] Seli E, Sakkas D, Scott R, Kwok SC, Rosendahl SM, Burns DH 2007 Noninvasive metabolomic profiling of embryo culture media using Raman and near-infrared spectroscopy correlates with reproductive potential of embryos in women undergoing in vitro fertilization. Fertil. Steril. 88, 1350–1357. (10.1016/j.fertnstert.2007.07.1390) 17923129

[20] Ding J, Xu T, Tan X, Jin H, Shao J, Li H 2017 Raman spectrum: a potential biomarker for embryo assessment during in vitro fertilization. Exp. Ther. Med. 13, 1789–1792. (10.3892/etm.2017.4160) 28565768 PMC5443171

[21] Cortezzi SS et al 2013 Prediction of embryo implantation potential by mass spectrometry fingerprinting of the culture medium. Reproduction 145, 453–462. (10.1530/REP-12-0168) 23404850

[22] Seli E, Botros L, Sakkas D, Burns DH 2008 Noninvasive metabolomic profiling of embryo culture media using proton nuclear magnetic resonance correlates with reproductive potential of embryos in women undergoing in vitro fertilization. Fertil. Steril. 90, 2183–2189. (10.1016/j.fertnstert.2008.07.1739) 18842260

[23] Pudakalakatti SM, Uppangala S, D'Souza F, Kalthur G, Kumar P, Adiga SK, Atreya HS 2013 NMR studies of preimplantation embryo metabolism in human assisted reproductive techniques: a new biomarker for assessment of embryo implantation potential. NMR Biomed. 26, 20–27. (10.1002/nbm.2814) 22714820

[24] Bingol K, Brüschweiler R 2015 Two elephants in the room: new hybrid nuclear magnetic resonance and mass spectrometry approaches for metabolomics. Curr. Opin. Clin. Nutr. Metab. Care 18, 471–477. (10.1097/MCO.0000000000000206) 26154280 PMC4533976

[25] Emwas AM 2015 The strengths and weaknesses of NMR spectroscopy and mass spectrometry with particular focus on metabolomics research. New York, NY: Springer. 10.1007/978-1-4939-2377-9_13 25677154

[26] Bingol K, Brüschweiler R 2017 Knowns and unknowns in metabolomics identified by multidimensional NMR and hybrid MS/NMR methods. Curr. Opin. Biotechnol. 43, 17–24. (10.1016/j.copbio.2016.07.006) 27552705 PMC5305439

[27] Pauli GF, Gödecke T, Jaki BU, Lankin DC 2012 Quantitative 1H NMR. Development and potential of an analytical method: an update. J. Nat. Prod. 75, 834–851. (10.1021/np200993k) 22482996 PMC3384681

[28] Markley JL, Brüschweiler R, Edison AS, Eghbalnia HR, Powers R, Raftery D, Wishart DS 2017 The future of NMR-based metabolomics. Curr. Opin. Biotechnol. 43, 34–40. (10.1016/j.copbio.2016.08.001) 27580257 PMC5305426

[29] Zhao Y et al 2012 Metabolic profiles characterizing different phenotypes of polycystic ovary syndrome: plasma metabolomics analysis. BMC Med. 10, 153–175. (10.1186/1741-7015-10-153) 23198915 PMC3599233

[30] RoyChoudhury S et al 2016 Serum metabolomics of Indian women with polycystic ovary syndrome using 1H NMR coupled with a pattern recognition approach. Mol. Biosyst. 12, 3407–3416. (10.1039/c6mb00420b) 27714060

[31] Bavister BD 2002 How animal embryo research led to the first documented human IVF. Reprod. Biomed. Online 4, 24–29. (10.1016/S1472-6483(12)60008-X) 12470332

[32] Nagana Gowda GA, Raftery D 2015 Can NMR solve some significant challenges in metabolomics? J. Magn. Reson. 260, 144–160. (10.1016/j.jmr.2015.07.014) 26476597 PMC4646661

[33] Fan TWM, Lane AN 2016 Applications of NMR spectroscopy to systems biochemistry. Prog. Nucl. Magn. Reson. Spectrosc. 92–93, 18–53. (10.1016/j.pnmrs.2016.01.005) PMC4850081 26952191

[34] Takis PG, Ghini V, Tenori L, Turano P, Luchinat C 2019 Uniqueness of the NMR approach to metabolomics. Trends Anal. Chem. 120, 115 300–115 308. (10.1016/j.trac.2018.10.036)

[35] Emwas AH et al 2019 NMR spectroscopy for metabolomics research. Metabolites 9, 123–162. (10.3390/metabo9070123) PMC6680826 31252628

[36] Wong KC 2014 Review of NMR spectroscopy: basic principles, concepts and applications in chemistry. J. Chem. Educ. 91, 1103–1104. (10.1021/ed500324w)

[37] Mishkovsky M, Frydman L 2009 Principles and progress in ultrafast multidimensional nuclear magnetic resonance. Annu. Rev. Phys. Chem. 60, 429–448. (10.1146/annurev.physchem.040808.090420) 18999994

[38] Wishart DS 2008 Quantitative metabolomics using NMR. Trends Anal. Chem. 27, 228–237. (10.1016/j.trac.2007.12.001)

[39] Barding GA, Salditos R, Larive CK 2012 Quantitative NMR for bioanalysis and metabolomics. Anal. Bioanal. Chem. 404, 1165–1179. (10.1007/s00216-012-6188-z) 22766756

[40] Nargund S, Joffe ME, Tran D, Tugarinov V, Sriram G 2013 Nuclear magnetic resonance methods for metabolic fluxomics. Totowa, NJ: Humana Press. 10.1007/978-1-62703-299-5_16 23417811

[41] Truong QX, Yoon JM, Shanks JV 2014 Isotopomer measurement techniques in metabolic flux analysis I: nuclear magnetic resonance (10.1007/978-1-62703-661-0) 24218211

[42] Serra O, Chatterjee S, Huang W, Stark RE 2012 Mini-review: What nuclear magnetic resonance can tell us about protective tissues. Plant Sci. 195, 120–124. (10.1016/j.plantsci.2012.06.013) 22921005 PMC3428714

[43] Moser E, Laistler E, Schmitt F, Kontaxis G 2017 Ultra-high field NMR and MRI—the role of magnet technology to increase sensitivity and specificity. Front. Phys. 5, 1–15. (10.3389/fphy.2017.00033)

[44] Schlotterbeck G, Ross A, Hochstrasser R, Senn H, Kuhn T, Marek D, Schett O 2002 High-resolution capillary tube NMR: a miniaturized 5-µL high-sensitivity TXI probe for mass-limited samples, off-line LC NMR, and HT. Anal. Chem. 74, 4464–4471. (10.1021/ac025556e) 12236357

[45] Martin GE. (2005).

[46] Grimes JH, Connell TMO 2011 The application of micro-coil NMR probe technology to metabolomics of urine and serum. J. Biomol. NMR 49, 297–305. (10.1007/s10858-011-9488-2) 21380855

[47] Nagato EG, Lankadurai BP, Soong R, Simpson AJ, Simpson MJ 2015 Development of an NMR microprobe procedure for high-throughput environmental metabolomics of Daphnia magna. Magn. Reson. Chem. 53, 745–753. (10.1002/mrc.4236) 25891518

[48] Balaban B et al 2011 The Istanbul consensus workshop on embryo assessment: proceedings of an expert meeting. Hum. Reprod. 26, 1270–1283. (10.1093/humrep/der037) 21502182

[49] Friebolin H 2011 Basic one- and two-dimensional NMR spectroscopy, 5th edn New York, NY: Wiley-VCH.

[50] Günther H 2013 NMR spectroscopy: basic principles, concepts and applications in chemistry. New York, NY: Wiley-VCH.

[51] Keeler J 2010 Understanding NMR spectroscopy. New York, NY: Wiley.

[52] Weljie AM, Newton J, Mercier P, Carlson E, Slupsky CM 2006 Targeted profiling: quantitative analysis of 1H NMR metabolomics data. Anal. Chem. 78, 4430–4442. (10.1021/ac060209g) 16808451

[53] Graham SF, Ruiz-Aracama A, Lommen A, Cannizzo FT, Biolatti B, Elliott CT, Mooney MH 2012 Use of NMR metabolomic plasma profiling methodologies to identify illicit growth-promoting administrations. Anal. Bioanal. Chem. 403, 573–582. (10.1007/s00216-012-5815-z) 22370585

[54] Nikolaev Y, Ripin N, Soste M, Picotti P, Iber D, Allain FHT 2019 Systems NMR: single-sample quantification of RNA, proteins and metabolites for biomolecular network analysis. Nat. Methods 16, 743–749. (10.1038/s41592-019-0495-7) 31363225 PMC6837886

[55] Giraudeau P, Frydman L 2014 Ultrafast 2D NMR: an emerging tool in analytical spectroscopy. Annu. Rev. Anal. Chem. 7, 129–161. (10.1146/annurev-anchem-071213-020208) PMC5040491 25014342

[56] Wishart DS et al 2009 HMDB: a knowledgebase for the human metabolome. Nucleic Acids Res. 37, D603–D610. (10.1093/nar/gkn810) 18953024 PMC2686599

[57] Cui Q et al 2008 Metabolite identification via the Madison Metabolomics Consortium Database. Nat. Biotechnol. 26, 162–164. (10.1038/nbt0208-162) 18259166

[58] Kuhn S, Schlörer NE 2015 Facilitating quality control for spectra assignments of small organic molecules: Nmrshiftdb2—a free in-house NMR database with integrated LIMS for academic service laboratories. Magn. Reson. Chem. 53, 582–589. (10.1002/mrc.4263) 25998807

[59] Ulrich EL et al 2007 BioMagResBank. Nucleic Acids Res. 36, D402–D408. (10.1093/nar/gkm957) 17984079 PMC2238925

[60] Wishart DS et al 2007 HMDB: the human metabolome database. Nucleic Acids Res. 35, 521–526. (10.1093/nar/gkl923) PMC1899095 17202168

[61] Hao J, Astle W, De Iorio M, Ebbels TMD 2012 BATMAN—an R package for the automated quantification of metabolites from nuclear magnetic resonance spectra using a Bayesian model. Bioinformatics 28, 2088–2090. (10.1093/bioinformatics/bts308) 22635605

[62] Ravanbakhsh S et al 2015 Accurate, fully-automated NMR spectral profiling for metabolomics. PLoS ONE 10, e0124219–e0124234. (10.1371/journal.pone.0124219) 26017271 PMC4446368

[63] Xia J, Bjorndahl TC, Tang P, Wishart DS 2008 MetaboMiner—semi-automated identification of metabolites from 2D NMR spectra of complex biofluids. BMC Bioinformatics 9, 507 (10.1186/1471-2105-9-507) 19040747 PMC2612014

[64] Johnson SR, Lange BM 2015 Open-access metabolomics databases for natural product research: present capabilities and future potential. Front. Bioeng. Biotechnol. 3, 1–10. (10.3389/fbioe.2015.00022) 25789275 PMC4349186

[65] Dubey A, Rangarajan A, Pal D, Atreya HS 2015 Pattern recognition-based approach for identifying metabolites in nuclear magnetic resonance-based metabolomics. Anal. Chem. 87, 7148–7155. (10.1021/acs.analchem.5b00990) 26101967

[66] Dubey A, Rangarajan A, Pal D, Atreya HS 2015 Chemical shifts to metabolic pathways: identifying metabolic pathways directly from a single 2D NMR spectrum. Anal. Chem. 87, 12 197–12 205. (10.1021/acs.analchem.5b03082) 26556218

[67] Hyberts SG et al 2007 Ultrahigh-resolution 1H−13C HSQC spectra of metabolite mixtures using nonlinear sampling and forward maximum entropy reconstruction. J. Am. Chem. Soc. 129, 5108–5116. (10.1021/ja068541x) 17388596 PMC2631400

[68] Ludwig C, Ward DG, Martin A, Viant MR, Ismail T, Johnson PJ, Wakelam MJO, Günther UL 2009 Fast targeted multidimensional NMR metabolomics of colorectal cancer. Magn. Reson. Chem. 47, S68–S73. (10.1002/mrc.2519) 19790200

[69] Giraudeau P, Massou S, Robin Y, Cahoreau E, Portais J-C, Akoka S 2011 Ultrafast quantitative 2D NMR: an efficient tool for the measurement of specific isotopic enrichments in complex biological mixtures. Anal. Chem. 83, 3112–3119. (10.1021/ac200007p) 21417426

[70] Bingol K, Brüschweiler R 2014 Multidimensional approaches to NMR-based metabolomics. Anal. Chem. 86, 47–57. (10.1021/ac403520j) 24195689 PMC4467887

[71] Le Guennec A, Giraudeau P, Caldarelli S 2014 Evaluation of fast 2D NMR for metabolomics. Anal. Chem. 86, 5946–5954. (10.1021/ac500966e) 24856256

[72] Pudakalakatti SM, Dubey A, Jaipuria G, Shubhashree U, Adiga SK, Moskau D, Atreya HS 2014 A fast NMR method for resonance assignments: application to metabolomics. J. Biomol. NMR 58, 165–173. (10.1007/s10858-014-9814-6) 24488481

[73] Pudakalakatti SM, Dubey A, Atreya HS 2015 Simultaneous acquisition of three NMR spectra in a single experiment for rapid resonance assignments in metabolomics. J. Chem. Sci. 127, 1091–1097. (10.1007/s12039-015-0868-0)

[74] Gierth P, Codina A, Schumann F, Kovacs H, Kupče E 2015 Fast experiments for structure elucidation of small molecules: Hadamard NMR with multiple receivers. Magn. Reson. Chem. 53, 940–944. (10.1002/mrc.4292) 26302997

[75] Singh A, Dubey A, Adiga SK, Atreya HS 2018 Phase modulated 2D HSQC-TOCSY for unambiguous assignment of overlapping spin systems. J. Magn. Reson. 286, 10–16. (10.1016/j.jmr.2017.11.005) 29169027

[76] Von Schlippenbach T, Oefner PJ, Gronwald W 2018 Systematic evaluation of non-uniform sampling parameters in the targeted analysis of urine metabolites by 1H, 1H 2D NMR spectroscopy. Sci. Rep. 8, 4249 (10.1038/s41598-018-22541-0) 29523811 PMC5844889

[77] Barnwal RP, Rout AK, Atreya HS, Chary KVR 2008 Identification of C-terminal neighbours of amino acid residues without an aliphatic 13C*γ* as an aid to NMR assignments in proteins. J. Biomol. NMR 41, 191–197. (10.1007/s10858-008-9254-2) 18633715

[78] Atreya HS 2012 Isotope labeling in biomolecular NMR, 1st edn Dordrecht, The Netherlands: Springer.

[79] Mulleti S, Singh A, Brahmkhatri VP, Chandra K, Mukherjee SP, Seelamantula CS, Atreya HS 2017 Super-resolved nuclear magnetic resonance spectroscopy. Sci. Rep. 7, 1–10. (10.1038/s41598-017-09884-w) 28851979 PMC5575056

[80] Patel AB, Srivastava S, Phadke RS, Govil G 1998 Arginine activates glycolysis of goat epididymal spermatozoa: an NMR study. Biophys. J. 75, 1522–1528. (10.1016/S0006-3495(98)74071-8) 9726954 PMC1299827

[81] Inoue H, Yoshioka T 1980 Measurement of intracellular pH in sea urchin eggs by 31P NMR. J. Cell. Physiol. 105, 461–468. (10.1002/jcp.1041050310) 6780576

[82] Colman A, Gadian DG 1976 31P nuclear-magnetic-resonance studies on the developing embryos of *Xenopus laevis*. Eur. J. Biochem. 61, 387–396. (10.1111/j.1432-1033.1976.tb10032.x) 1248465

[83] Rubessa M, Ambrosi A, Gonzalez D, Kathryn P, Matthew MP 2018 Non-invasive nuclear magnetic resonance analysis of male and female embryo metabolites during in vitro embryo culture. Metabolomics 14, 1–9. (10.1007/s11306-018-1414-0) 30830365

[84] Kirkegaard K Svane ASP, Nielsen JS, HindkjæR JJ, Nielsen NC, Ingerslev HJ 2014 Nuclear magnetic resonance metabolomic profiling of Day 3 and 5 embryo culture medium does not predict pregnancy outcome in good prognosis patients: a prospective cohort study on single transferred embryos. Hum. Reprod. 29, 2413–2420. (10.1093/humrep/deu236) 25256566

[85] Khosravi P et al 2019 Deep learning enables robust assessment and selection of human blastocysts after in vitro fertilization. npj Digit. Med. 2, 1–9. (10.1038/s41746-019-0096-y) 31304368 PMC6550169

[86] Hardy K, Hooper MAK, Handyside AH, Rutherford AJ, Winston RML, Leese HJ 1989 Non-invasive measurement of glucose and pyrovate uptake by individual human oocytes and preimplantation embryos. Hum. Reprod. 4, 348 (10.1093/oxfordjournals.humrep.a136905) 2918073

[87] Vergouw CG, Kieslinger DC, Kostelijk EH, Botros LL, Schats R, Hompes PG, Sakkas D, Lambalk CB 2012 Day 3 embryo selection by metabolomic profiling of culture medium with near-infrared spectroscopy as an adjunct to morphology: a randomized controlled trial. Hum. Reprod. 27, 2304–2311. (10.1093/humrep/des175) 22647453

[88] Liang B et al 2019 Raman profiling of embryo culture medium to identify aneuploid and euploid embryos. Fertil. Steril. 111, 753–762.e1. (10.1016/j.fertnstert.2018.11.036) 30683589

[89] Conaghan J, Hardy K, Handyside AH, Winston RML, Leese HJ 1993 Selection criteria for human embryo transfer: a comparison of pyruvate uptake and morphology. J. Assist. Reprod. Genet. 10, 21–30. (10.1007/BF01204436) 8499675

[90] Gardner DK, Lane M, Stevens J, Schoolcraft WB 2001 Noninvasive assessment of human embryo nutrient consumption as a measure of developmental potential. Fertil. Steril. 76, 1175–1180. (10.1016/S0015-0282(01)02888-6) 11730746

[91] Brison DR, Houghton FD, Falconer D, Roberts SA, Hawkhead J, Humpherson PG, Lieberman BA, Leese HJ 2004 Identification of viable embryos in IVF by non-invasive measurement of amino acid turnover. Hum. Reprod. 19, 2319–2324. (10.1093/humrep/deh409) 15298971

[92] Seli E, Vergouw CG, Morita H, Botros L, Roos P, Lambalk CB, Yamashita N, Kato O, Sakkas D 2010 Noninvasive metabolomic profiling as an adjunct to morphology for noninvasive embryo assessment in women undergoing single embryo transfer. Fertil. Steril. 94, 535–542. (10.1016/j.fertnstert.2009.03.078) 19589524

[93] D'Souza F, Uppangala S, Asampille G, Salian SR, Kalthur G, Talevi R, Atreya HS, Adiga SK 2018 Spent embryo culture medium metabolites are related to the in vitro attachment ability of blastocysts. Sci. Rep. 8, 1–10. (10.1038/s41598-018-35342-2) 30451915 PMC6242932

[94] Steptoe PC, Edwards RG 1978 Birth after the reimplantation of a human embryo. Arch. Pathol. Lab. Med. 116, 321 (10.1016/s0140-6736(78)92957-4) 79723

[95] Lane M, Gardner DK 2007 Embryo culture medium: which is the best? Best Pract. Res. Clin. Obstet. Gynaecol. 21, 83–100. (10.1016/j.bpobgyn.2006.09.009) 17090393

[96] Houghton FD 2003 Non-invasive amino acid turnover predicts human embryo developmental capacity. Hum. Reprod. 18, 1756–1757. (10.1093/humrep/deg389) 11925397

[97] Cimadomo D et al 2018 Continuous embryo culture elicits higher blastulation but similar cumulative delivery rates than sequential: a large prospective study. J. Assist. Reprod. Genet. 35, 1329–1338. (10.1007/s10815-018-1195-4) 29725911 PMC6063814

[98] Sunde A, Brison D, Dumoulin J, Harper J, Lundin K, Magli MC, Van Den Abbeel E, Veiga A. 2016 Time to take human embryo culture seriously. Hum. Reprod. 31, 2174–2182. (10.1093/humrep/dew157) 27554442

[99] Simopoulou M et al 2018 Considerations regarding embryo culture conditions: from media to epigenetics. In Vivo 32, 451–460. (10.21873/invivo.11261) 29695546 PMC6000787

[100] Xella S, Marsella T, Tagliasacchi D, Giulini S, La Marca A, Tirelli A, Volpe A. 2010 Embryo quality and implantation rate in two different culture media: ISM1 versus Universal IVF Medium. Fertil. Steril. 93, 1859–1863. (10.1016/j.fertnstert.2008.12.030) 19152877

[101] Morbeck DE, Baumann NA, Oglesbee D 2017 Composition of single-step media used for human embryo culture. Fertil. Steril. 107, 1055–1060; e1. (10.1016/j.fertnstert.2017.01.007) 28238490

[102] Tarahomi M, Vaz FM, Van Straalen JP, Schrauwen FAP, van Wely M, Hamer G, Repping S, Mastenbroek S 2019 The composition of human preimplantation embryo culture media and their stability during storage and culture. Hum. Reprod. 34, 1450–1461. (10.1093/humrep/dez102) 31348827

[103] Marhuenda-Egea FC, Gonsálvez-Álvarez R, Martínez-Sabater E, Lledó B, Ten J, Bernabeu R 2011 Improving human embryos selection in IVF: non-invasive metabolomic and chemometric approach. Metabolomics 7, 247–256. (10.1007/s11306-010-0245-4)

[104] Rinaudo P et al 2012 1H NMR based profiling of spent culture media cannot predict success of implantation for day 3 human embryos. J. Assist. Reprod. Genet. 29, 1435–1442. (10.1007/s10815-012-9877-9) 23090745 PMC3528870

[105] Sánchez-Ribas I, Riqueros M, Vime P, Puchades-Carrasco L, Jönsson T, Pineda-Lucena A, Ballesteros A, Domínguez F, Simón C 2012 Differential metabolic profiling of non-pure trisomy 21 human preimplantation embryos. Fertil. Steril. 98, 1157–1164.e2. (10.1016/j.fertnstert.2012.07.1145) 22959456

[106] Nadal-Desbarats L, Veau S, Blasco H, Emond P, Royere D, Andres CR, Guérif F 2013 Is NMR metabolic profiling of spent embryo culture media useful to assist in vitro human embryo selection? Magn. Reson. Mater. Phys. Biol. Med. 26, 193–202. (10.1007/s10334-012-0331-x) 22878530

[107] Wallace M, Cottell E, Cullinane J, McAuliffe FM, Wingfield M, Brennan L 2014 1H NMR based metabolic profiling of day 2 spent embryo media correlates with implantation potential. Syst. Biol. Reprod. Med. 6368, 58–63. (10.3109/19396368.2013.854426) 24261874

[108] Uppangala S, D'Souza F, Pudakalakatti S, Atreya HS, Raval K, Kalthur G, Adiga SK 2016 Laser assisted zona hatching does not lead to immediate impairment in human embryo quality and metabolism. Syst. Biol. Reprod. Med. 62, 396–403. (10.1080/19396368.2016.1217952) 27598006

[109] Uppangala S, Pudakalakatti S, D'souza F, Salian SR, Kalthur G, Kumar P, Atreya HS, Adiga SK 2016 Influence of sperm DNA damage on human preimplantation embryo metabolism. Reprod. Biol. 16, 234–241. (10.1016/j.repbio.2016.07.004) 27492188

[110] Rubessa M, Ambrosi A, Gonzalez-Pena D, Polkoff KM, Denmark SE, Wheeler MB 2016 Non-invasive analysis of bovine embryo metabolites during in vitro embryo culture using nuclear magnetic resonance. AIMS Bioeng. 3, 538–551. (10.3934/bioeng.2016.4.538) 30830365

[111] Perkel KJ, Madan P 2017 Spent culture medium analysis from individually cultured bovine embryos demonstrates metabolomic differences. Zygote 25, 662–674. (10.1017/S0967199417000417) 29032784

[112] D'Souza F et al 2016 Unraveling the association between genetic integrity and metabolic activity in pre-implantation stage embryos. Sci. Rep. 6, 37291 (10.1038/srep37291) 27853269 PMC5112559

[113] D'Souza F, Asampille G, Uppangala S, Kalthur G, Atreya HS, Adiga SK 2019 Sperm-mediated DNA lesions alter metabolite levels in spent embryo culture medium. Reprod. Fertil. Dev. 31, 443–450. (10.1071/RD18136) 30223941

[114] Piñero-Sagredo E, Nunes S, De Los Santos MJ, Celda B, Esteve V. 2010 NMR metabolic profile of human follicular fluid. NMR Biomed. 23, 485–495. (10.1002/nbm.1488) 20336675

[115] Gérard N, Fahiminiya S, Grupen CG, Nadal-Desbarats L 2015 Reproductive physiology and ovarian folliculogenesis examined via 1H-NMR metabolomics signatures: a comparative study of large and small follicles in three mammalian species (*Bos taurus*, *Sus scrofa domesticus* and *Equus ferus caballus*). Omi. A J. Integr. Biol. 19, 31–40. (10.1089/omi.2014.0097) 25393852

[116] Wallace M, Cottell E, Gibney MJ, McAuliffe FM, Wingfield M, Brennan L 2012 An investigation into the relationship between the metabolic profile of follicular fluid, oocyte developmental potential, and implantation outcome. Fertil. Steril. 97, 1078–1084. (10.1016/j.fertnstert.2012.01.122) 22365382

[117] McRae C, Baskind NE, Orsi NM, Sharma V, Fisher J 2012 Metabolic profiling of follicular fluid and plasma from natural cycle in vitro fertilization patients—a pilot study. Fertil. Steril. 98, 1449–1457.e6. (10.1016/j.fertnstert.2012.07.1131) 22921074

[118] Zhang Y, Liu L, Yin TL, Yang J, Xiong CL 2017 Follicular metabolic changes and effects on oocyte quality in polycystic ovary syndrome patients. Oncotarget 8, 80 472–80 480. (10.18632/oncotarget.19058) PMC5655213 29113318

[119] Jaccarino N, Amato J, Pagano B, D'Oriano L 2019 1H NMR-based metabolomics study on follicular fluid from patients with polycystic ovary syndrome. Biochim. Clin. 43, 256–263. (10.19186/BC)

[120] Marianna SM et al 2017 Metabolomic profiling and biochemical evaluation of the follicular fluid of endometriosis patients. Mol. Biosyst. 13, 1213–1222. (10.1039/c7mb00181a) 28475193

[121] Castiglione Morelli MA, Iuliano A, Schettini SCA, Petruzzi D, Ferri A, Colucci P, Viggiani L, Cuviello F, Ostuni A 2018 NMR metabolomics study of follicular fluid in women with cancer resorting to fertility preservation. J. Assist. Reprod. Genet. 35, 2063–2070. (10.1007/s10815-018-1281-7) 30069850 PMC6240554

[122] Karaer A, Tuncay G, Mumcu A, Dogan B 2019 Metabolomics analysis of follicular fluid in women with ovarian endometriosis undergoing in vitro fertilization. Syst. Biol. Reprod. Med. 65, 39–47. (10.1080/19396368.2018.1478469) 29806498

[123] Castiglione Morelli MA, Iuliano A, Schettini SCA, Petruzzi D, Ferri A, Colucci P, Viggiani L, Cuviello F, Ostuni A 2019 NMR metabolic profiling of follicular fluid for investigating the different causes of female infertility: a pilot study. Metabolomics 15, 1–10. (10.1007/s11306-019-1481-x) 30830455

[124] Gosden RG, Sadler IH, Reed D, Hunter RHF 1990 Characterization of ovarian follicular fluids of sheep, pigs and cows using proton nuclear magnetic resonance spectroscopy. Experientia 46, 1012–1015. (10.1007/BF01940658) 2171975

[125] Gérard N, Loiseau S, Duchamp G, Seguin F 2002 Analysis of the variations of follicular fluid composition during follicular growth and maturation in the mare using proton nuclear magnetic resonance (1H NMR). Reproduction 124, 241–248. (10.1530/rep.0.1240241) 12141937

[126] Bertoldo MJ, Nadal-Desbarats L, Gérard N, Dubois A, Holyoake PK, Grupen CG 2013 Differences in the metabolomic signatures of porcine follicular fluid collected from environments associated with good and poor oocyte quality. Reproduction 146, 221–231. (10.1530/REP-13-0142) 23801780

[127] RoyChoudhury S, Singh A, Gupta NJ, Srivastava S, Joshi MV., Chakravarty B, Chaudhury K 2016 Repeated implantation failure versus repeated implantation success: discrimination at a metabolomic level. Hum. Reprod. 31, 1265–1274. (10.1093/humrep/dew064) 27060172

[128] Lyman JT, Rubessa M, Oh DK, Ginger L, Magin RL, Wheeler MB 2016 Investigation into the use of nuclear magnetic resonance spectroscopy for viability predictions, in real time on mouse preimplantation embryos. Int. J. New Technol. Res. 2, 14–20.

[129] Bodart JF, Wieruszeski JM, Amniai L, Leroy A, Landrieu I, Rousseau-Lescuyer A, Vilain JP, Lippens G 2008 NMR observation of Tau in *Xenopus* oocytes. J. Magn. Reson. 192, 252–257. (10.1016/j.jmr.2008.03.006) 18378475

[130] Zhao SJ, Tian JS, Tai G, Gao XX, Liu HL, Du GH, Liu XJ, Qin XM 2019 1H NMR-based metabolomics revealed the protective effects of Guilingji on the testicular dysfunction of aging rats. J. Ethnopharmacol. 238, 111839 (10.1016/j.jep.2019.111839) 30928501

[131] Chew WM, Hricak H, McClure RD, Wendland FM. 1990 In vivo human testicular function assessed with P-31 MR spectroscopy. Radiology 177, 743–747. 2243981 10.1148/radiology.177.3.2243981

[132] Griffin JLL, Troke J, Walker LAA, Shore RFF, Lindon JCC, Nicholson JKK 2000 The biochemical profile of rat testicular tissue as measured by magic angle spinning 1H NMR spectroscopy. FEBS Lett. 486, 225–229. (10.1016/S0014-5793(00)02307-3) 11119708

[133] Jarak I, Almeida S, Carvalho RA, Sousa M, Barros A, Alves MG, Oliveira PF 2018 Senescence and declining reproductive potential: Insight into molecular mechanisms through testicular metabolomics. BBA—Mol. Basis Dis. 1864, 3388–3396. (10.1016/j.bbadis.2018.07.028) 30059728

[134] Lin CY, Hung PH, VandeVoort CA, Miller MG 2009 1H NMR to investigate metabolism and energy supply in rhesus macaque sperm. Reprod. Toxicol. 28, 75–80. (10.1016/j.reprotox.2009.03.005) 19490998

[135] Paiva C, Amaral A, Rodriguez M, Canyellas N, Correig X, Ballescà JL, Ramalho-Santos J, Oliva R 2015 Identification of endogenous metabolites in human sperm cells using proton nuclear magnetic resonance (1H-NMR) spectroscopy and gas chromatography-mass spectrometry (GC-MS). Andrology 3, 496–505. (10.1111/andr.12027) 25854681

[136] Reynolds S, Fadhlina Bt Ismail N, Calvert SJ, Pacey AA, Paley MNJ 2017 Evidence for rapid oxidative phosphorylation and lactate fermentation in motile human sperm by hyperpolarized 13C magnetic resonance spectroscopy Sci. Rep. 7, 1–8. (10.1038/s41598-017-04146-1) 28659585 PMC5489489

[137] Reynolds S, Calvert SJ, Paley MN, Pacey AA 2017 1H magnetic resonance spectroscopy of live human sperm. Mol. Hum. Reprod. 23, 441–451. (10.1093/molehr/gax025) 28431025 PMC5909857

[138] Calvert SJ, Reynolds S, Paley MN, Walters SJ, Pacey AA 2018 Probing human sperm metabolism using 13C-magnetic resonance spectroscopy. Mol. Hum. Reprod. 25, 30–41. (10.1093/molehr/gay046) PMC6314230 30395244

[139] Risten RC, Schackmann RW, Dahlquist FW, Shapiro BM, Christen R, Schackmann RW, Dahlquist FW, Shapiro BM 1983 31P-NMR analysis of sea urchin sperm activation: reversible formation of high energy phosphate compounds by changes in intracellular pH. Exp. Cell Res. 149, 289–294. (10.1016/0014-4827(83)90400-7) 6641799

[140] Robitaille PA, Robitaille P-ML, Brown GG 1986 31P-NMR studies of *Limulus polyphemus*: spermatozoa at rest and after motility. J. Exp. Zool. 238, 89–98. (10.1002/jez.1402380111)

[141] Dreanno C, Seguin F, Cosson J, Suquet M, Billard R 2000 1H-NMR and 31P-NMR analysis of energy metabolism of quiescent and motile turbot (*Psetta maxima*) spermatozoa. J. Exp. Zool. 286, 513–522. (10.1002/(SICI)1097-010X(20000401)286:5<513::AID-JEZ9>3.0.CO;2-5) 10684575

[142] Robitaille P-ML, Mumford KG, Brown GG 1987 31P nuclear magnetic resonance study of trout spermatozoa at rest, after motility, and during short-term storage. Biochem. Cell Biol. 65, 474–485. (10.1139/o87-061)

[143] Smith MB, Babcock DF, Lardy HA 1985 A 31P NMR study of the epididymis and epididymal sperm of the bull and hamster. Biol. Reprod. 33, 1029–1040. (10.1095/biolreprod33.5.1029) 4074801

[144] Lynch MJ, Masters J, Pryor JP, Lindon JC, Spraul M, Foxall PJD, Nicholson JK 1994 Ultra high field NMR spectroscopic studies on human seminal fluid, seminal vesicle and prostatic secretions. J. Pharm. Biomed. Anal. 12, 5–19. (10.1016/0731-7085(94)80004-9) 8161606

[145] Hamamah S, Seguin F, Bujan L, Barthelemy C, Mieusset R, Lansac J 1998 Quantification by magnetic resonance spectroscopy of metabolites in seminal plasma able to differentiate different forms of azoospermia. Hum. Reprod. 13, 132–135. (10.1093/humrep/13.1.132) 9512244

[146] Tomlins AM, Foxall PJ d, Lynch MJ, Parkinson J, Everett JR, Nicholson JK 1998 High resolution 1H NMR spectroscopic studies on dynamic biochemical processes in incubated human seminal fluid samples. Biochim. Biophys. Acta—Gen. Subj. 1379, 367–380. (10.1016/S0304-4165(97)00116-5) 9545599

[147] Sharma U, Chaudhury K, Jagannathan NR, Guha SK 2001 A proton NMR study of the effect of a new intravasal injectable male contraceptive RISUG on seminal plasma metabolites. Reproduction 122, 431–436. (10.1530/rep.0.1220431) 11597307

[148] Gupta A, Mahdi AA, Ahmad MK, Shukla KK, Bansal N, Jaiswer SP, Shankhwar SN 2011 A proton NMR study of the effect of Mucuna pruriens on seminal plasma metabolites of infertile males. J. Pharm. Biomed. Anal. 55, 1060–1066. (10.1016/j.jpba.2011.03.010) 21459537

[149] Gupta A, Mahdi AA, Ahmad MK, Shukla KK, Jaiswer SP, Shankhwar SN 2011 1H NMR spectroscopic studies on human seminal plasma: a probative discriminant function analysis classification model. J. Pharm. Biomed. Anal. 54, 106–113. (10.1016/j.jpba.2010.07.021) 20719458

[150] Gupta A, Mahdi AA, Shukla KK, Ahmad MK, Bansal N, Sankhwar P, Sankhwar SN 2013 Efficacy of *Withania somnifera* on seminal plasma metabolites of infertile males: a proton NMR study at 800 MHz. J. Ethnopharmacol. 149, 208–214. (10.1016/j.jep.2013.06.024) 23796876

[151] Bonechi C, Collodel G, Donati A, Martini S, Moretti E, Rossi C 2015 Discrimination of human semen specimens by NMR data, sperm parameters, and statistical analysis. Syst. Biol. Reprod. Med. 61, 353–359. (10.3109/19396368.2015.1054003) 26236922

[152] Zhang X, Diao R, Zhu X, Li Z, Cai Z 2015 Metabolic characterization of asthenozoospermia using nontargeted seminal plasma metabolomics. Clin. Chim. Acta 450, 254–261. (10.1016/j.cca.2015.09.001) 26342261

[153] Darbandi M et al 2019 Reactive oxygen species-induced alterations in H19-Igf2 methylation patterns, seminal plasma metabolites, and semen quality. J. Assist. Reprod. Genet. 36, 241–253. (10.1007/s10815-018-1350-y) 30382470 PMC6420547

[154] Mumcu A, Karaer A, Dogan B, Tuncay G 2019 Metabolomics analysis of seminal plasma in patients with idiopathic Oligoasthenoteratozoospermia using high-resolution NMR spectroscopy. Andrology 8, 450–456. (10.1111/andr.12707) 31520509

[155] Mehrparvar B, Chashmniam S, Nobakht F, Amini M, Javidi A, Minai-Tehrani A, Arjmand B, Gilany K 2020 Metabolic profiling of seminal plasma from teratozoospermia patients. J. Pharm. Biomed. Anal. 178, 112903 (10.1016/j.jpba.2019.112903) 31605879

[156] Ebrahimi F, Ibrahim B, Teh CH, Murugaiyah V, Chan KL 2017 NMR-based plasma metabolomic discrimination for male fertility assessment of rats treated with *Eurycoma longifolia* extracts. Syst. Biol. Reprod. Med. 63, 179–191. (10.1080/19396368.2017.1295332) 28306342

[157] Barberet J et al 2019 Can novel early non-invasive biomarkers of embryo quality be identified with time-lapse imaging to predict live birth? Hum. Reprod. 34, 1439–1449. (10.1093/humrep/dez085) 31287145 PMC6688874

[158] Mishkovsky M, Frydman L 2008 Progress in hyperpolarized ultrafast 2D NMR spectroscopy. ChemPhysChem 9, 2340–2348. (10.1002/cphc.200800461) 18850607

[159] Adams RW 2014 Pure shift nmr spectroscopy. In *eMagRes*, pp. 295–310. Chichester, UK: John Wiley & Sons.

[160] Zangger K 2015 Pure shift NMR. Prog. Nucl. Magn. Reson. Spectrosc. 86–87, 1–20. (10.1016/j.pnmrs.2015.02.002) 25919196

[161] Corcoran O, Spraul M, Corcoran O 2003 LC–NMR– MS in drug discovery. Drug Discov. Today 8, 624–631. 12867148 10.1016/s1359-6446(03)02749-1

[162] Walker GS, Connell TNO 2008 Comparison of LC-NMR and conventional NMR for structure elucidation in drug metabolism studies. Exp. Opin. Drug Metab. Toxicol. 4, 1295–1305. (10.1517/17425255.4.10.1295) 18798699

[163] Garrod S, Humpfer E, Spraul M, Connor SC, Polley S, Connelly J, Lindon JC, Nicholson JK, Holmes E 1999 High-resolution magic angle spinning 1H NMR spectroscopic studies on intact rat renal cortex and medulla. Magn. Reson. Med. 41, 1108–1118. (10.1002/(sici)1522-2594(199906)41:6<1108::aid-mrm6>3.0.co;2-m) 10371442

[164] Morvan D, Demidem A, Papon J, De Latour M, Claude Madelmont J. 2002 Melanoma tumors acquire a new phospholipid metabolism phenotype under cystemustine as revealed by high-resolution magic angle spinning proton nuclear magnetic resonance spectroscopy of intact tumor samples. Cancer Res. 62, 1890–1897. 11912170

[165] Hurd RE, Yen Y-F, Chen A. 2010 Hyperpolarized nuclear magnetic resonance spectroscopy: a new method for metabolomic research. In Methodologies for metabolomics (eds Lutz N, Sweedler J, Wevers R), pp. 446–471. Cambridge, UK: Cambridge University Press.

[166] Ardenkjaer-Larsen JH, Fridlund B, Gram A, Hansson G, Hansson L, Lerche MH, Servin R, Thaning M, Golman K. 2003 Increase in signal-to-noise ratio of >10 000 times in liquid-state NMR. Proc. Natl Acad. Sci. USA 100, 10 158–10 163. (10.1073/pnas.1733835100) 12930897 PMC193532

[167] Lerche MH, Karlsson M, Ardenkjær-Larsen JH, Jensen PR. 2019 Targeted metabolomics with quantitative dissolution dynamic nuclear polarization. In NMR-based metabolomics: methods and protocols (eds Gowda G, Raftery D), pp. 385–393. Cham, Switzerland: Springer. 10.1007/978-1-4939-9690-2_21 31463856

[168] Christensen CE, Karlsson M, Winther JR, Jensen PR, Lerche MH 2014 Non-invasive in-cell determination of free cytosolic [NAD+]/[NADH] ratios using hyperpolarized glucose show large variations in metabolic phenotypes. J. Biol. Chem. 289, 2344–2352. (10.1074/jbc.M113.498626) 24302737 PMC3900977

[169] Lin E 2012 Novel drug therapies and diagnostics for personalized medicine and nanomedicine in genome science, nanoscience, and molecular engineering. Pharm. Regul. Aff. Open Access 1, 1000e116–1000e117. (10.4172/2167-7689.1000e116)

